# Disruption of polyunsaturated fatty acid biosynthesis drives STING-dependent acute myeloid leukemia cell maturation and death

**DOI:** 10.1016/j.jbc.2024.107214

**Published:** 2024-03-22

**Authors:** Joice Kanefsky, Mary Basse, Judith Sokei, Orsola di Martino, Liana Valin, Yorrick Jaspers, Esteban Martinez, Jacklyn Huhn, Daniela Di Marcantonio, Jeffrey A. Magee, Aaron R. Goldman, Hsin-Yao Tang, Francesca Ferraro, Stephan Kemp, David L. Wiest, Stephen M. Sykes

**Affiliations:** 1 Fox Chase Cancer Center, Temple University Health System, Philadelphia, Pennsylvania, USA; 2 Department of Medicine, School of Medicine, Washington University in Saint Louis, St Louis, Missouri, USA; 3 Amsterdam University Medical Centers, Amsterdam, North Holland, Netherlands; 4 Proteomics & Metabolomics Facility, Wistar Institute, Philadelphia, Pennsylvania, USA

**Keywords:** FADS1, PUFA, AML, STING, phospholipid

## Abstract

The role of polyunsaturated fatty acid (PUFA) biosynthesis in acute myeloid leukemia (AML) remains largely undefined. A comparative expression analysis of 35 genes encoding fatty acid biosynthesis enzymes showed that fatty acid desaturase 1 (*FADS1*) was highly expressed across multiple AML subtypes relative to healthy controls and that elevated *FADS1* expression correlates with worse overall AML patient survival. Functionally, shRNA-mediated inhibition of FADS1 reduced AML cell growth *in vitro* and significantly delayed leukemia onset in an AML mouse model. AML cell lines depleted of FADS1 arrested in the G1/S-phase of the cell cycle, acquired characteristics of myeloid maturation and subsequently died. To understand the molecular consequences of FADS1 inhibition, a combination of mass spectrometry–based analysis of complex lipids and gene expression analysis (RNA-seq) was performed. FADS1 inhibition caused AML cells to exhibit significant lipidomic remodeling, including depletion of PUFAs from the phospholipids, phosphatidylserine, and phosphatidylethanolamine. These lipidomic alterations were accompanied by an increase induction of inflammatory and stimulator of interferon genes (STING)-mediated type-1 interferon signaling. Remarkably, genetic deletion of STING largely prevented the AML cell maturation and death phenotypes mediated by FADS1 inhibition. Highlighting the therapeutic implications of these findings, pharmacological blockade of PUFA biosynthesis reduced patient-derived AML cell numbers *ex vivo* but not that of healthy donor cells. Similarly, STING agonism attenuated patient-derived–AML survival; however, STING activation also reduced healthy granulocyte numbers. Collectively, these data unveil a previously unrecognized importance of PUFA biosynthesis in leukemogenesis and that imbalances in PUFA metabolism can drive STING-mediated AML maturation and death.

Fatty acid (FA) metabolism has emerged as a focal point of investigation in cancer metabolism (1, 2, 3). Free FAs and complex lipids support an assortment of cellular processes that are coopted in human cancer, such as energy metabolism (*i.e.*, FA oxidation), inflammation, and cell signaling. While the role of FA oxidation in acute myeloid leukemia (AML) has been investigated (4, 5) information on other aspects of FA metabolism, such as biosynthesis and desaturation, remains limited.

FAs are comprised of a long hydrocarbon (*i.e.*, acyl) chain attached to a C-terminal carboxyl group that exhibit extensive variability in chain length and degree of unsaturation (6). While mammalian cells can *de novo* synthesize saturated and monounsaturated fatty acids (MUFAs), polyunsaturated fatty acids (PUFAs) are solely acquired through diet through the consumption of omega-3 (ω-3) or omega-6 (ω-6) FAs. Once imported into cells, PUFA are further enzymatically modified (*e.g.*, alterations in chain length or the number of double bonds) to generate a variety of distinct PUFA that influence multiple cellular processes, such as inflammation as well as membrane composition and flexibility (7).

A key enzyme in the intracellular biosynthesis of PUFA is fatty acid desaturase 1 (FADS1), which desaturates both ω-3 and ω-6 FAs at the delta-5 (Δ5) position. FADS1 has gained attention in cancer biology as it is overexpressed in a variety of cancers and its levels are associated with tumor grade and prognosis (8, 9, 10). Furthermore, the product of FADS1 ω-6 desaturation, arachidonic acid (AA), has also been implicated in multiple human cancers, including AML where elevated plasma AA levels are associated with adverse risk (2, 11, 12). However, the role of FADS1 and its downstream product AA in AML pathogenesis has yet to be defined.

Stimulator of interferon genes (STING) is a transmembrane protein that resides in the endoplasmic reticulum (ER) membrane and is at the center of a powerful network of immune defense signaling cascades (13). Once activated, STING initiates IRF3-dependent type-1 interferon and NF-kB–mediated inflammatory signaling (13, 14) that can then induce a wide array of cellular responses, including cell death. Pharmacological activation of STING has shown antileukemia effects in mouse and human models of AML (15, 16, 17) and is currently being explored in clinical trials for a variety of blood and solid tumors (18).

Here, we show that disruptions in PUFA biosynthesis leads to activation of the STING pathway followed by AML cell death. Specifically, we show that the expression of key enzymes involved in PUFA biosynthesis, such as FADS1, are elevated across multiple AML subtypes and correlate with poorer outcomes. Additionally, shRNA-mediated inhibition of FADS1 is sufficient to impede cell cycling and causes AML cells to undergo maturation and subsequently die. Using a combination of transcriptomics and lipidomics, we demonstrate that FADS1 inhibition increased phospholipid saturation with a concomitant increase in STING-mediated type-1 interferon signaling. We also show that generic ablation of STING attenuates the antileukemic effects that stem from the imbalances in PUFA biosynthesis mediated either by *FADS1* knockdown or by pharmacologic attenuation of FA desaturation. Importantly, pharmacologic attenuation of FA desaturation also promotes maturation and death of patient-derived AML (PD-AML) cells without significant toxicity to healthy hematopoietic cells. Finally, STING agonism potently eliminates PD-AML cells. These data uncover a previously unrecognized metabolic dependency of AML cells with potential implications for treatments targeting FA metabolism.

## Results

### FADS1 expression is elevated across multiple AML subtypes and correlates with patient outcomes

**Figure 1:**
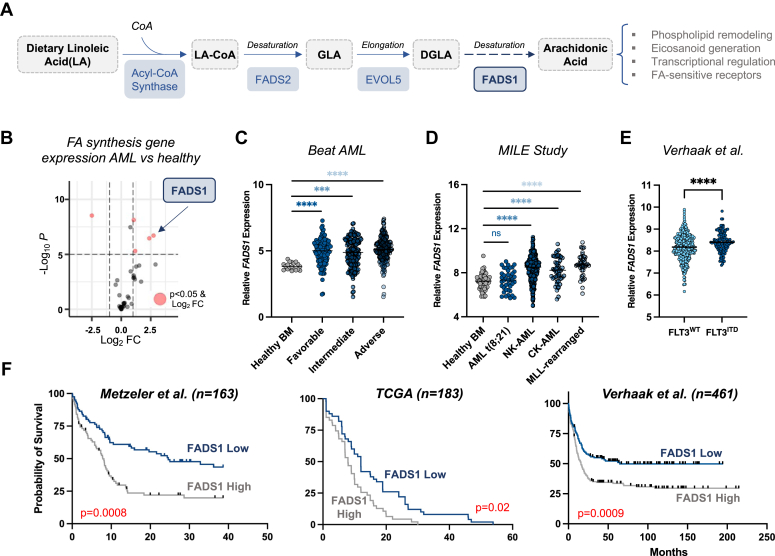
**FADS1 expression is elevated across multiple AML subtypes and correlates with patient outcomes.***A*, schematic detailing the steps during endogenous synthesis of AA and its fatty acyl-CoA, AA-CoA. The key enzymes are highlighted in *blue boxes*. *B*, *volcano plot* comparing the fold change in the expression of various fatty acid synthetic genes between AML patients and healthy BM populations from GSE9476. *C*, *dot plot* (extracted from Vizome, BeatAML) comparing the levels of FADS1 mRNA between BM cells from healthy donors and AML patient samples with the indicated genetic subtypes [Healthy BM (n = 19) *versus*: favorable (n = 120), ∗∗∗∗*p* < 0.0001; intermediate (n = 151), ∗∗∗*p* = 0.0001; adverse (n = 162), ∗∗∗∗*p* < 0.0001]. *D*, *dot plot* (extracted from Bloodspot (50) comparing the levels of FADS1 mRNA between BM cells from healthy donors and AML patient samples with the indicated genetic subtypes [Healthy BM (n = 73) *versus*: AML t(8;21) (n = 40), *p* < 0.55; NK-AML (n = 351), ∗∗∗∗*p* < 0.0001; CK-AML (n = 48), ∗∗∗∗*p* < 0.0001; *MLL*-rearranged (n = 39), ∗∗∗∗*p* < 0.0001]. *E*, comparison of FADS1 mRNA levels in FLT3^WT^ (n = 335) and FLT3^ITD^ (n = 126), ∗∗∗∗*p* < 0.0001 from Verhaak *et al.* (24) (data extracted from the Leukemia Gene Atlas (51)). *F*, Kaplan–Meier curve depicting the probability of survival of AML patients expressing FADS1 mRNA levels above (*gray line*) or below the median (*blue line*). Data for each of the three indicated datasets was extracted from the Leukemia Gene Atlas. AA-CoA, arachidonoyl-CoA; AML, acute myeloid leukemia; BM, bone marrow; CK-AML, complex-karyotype AML; DGLA, dihomo-gamma-linolenic acid; FADS1, fatty acid desaturase 1; GLA, gamma-linolenic acid; LA-CoA, linoleoyl-Co-A; *MLL*, mixed lineage leuekmia; NK-AML, normal-karyotype AML.

To investigate a potential role for FA biosynthesis in AML, we compared the expression of an annotated list of 35 FA biosynthetic-encoding genes (Table S1) in gene expression profiles (GEPs) from healthy donor hematopoietic cells (n = 38) and AML patients (n = 26) (19). We found that FADS1, an enzyme that catalyzes a rate-limiting step in PUFA biosynthesis (Fig. 1*A*), is significantly upregulated in AML patients (Fig. 1*B*). In two other AML patient cohorts (20, 21), we found that *FADS1* mRNA is elevated across multiple AML subtypes compared to healthy controls and is particularly high in mixed lineage leuekmia (*MLL*) (also known as *KMT2A*)-rearranged leukemia (Fig. 1, *C* and *D*). In addition, *FADS1* expression is significantly higher in AML samples bearing internal tandem duplications within the *FLT3* gene (FLT3^ITD^) than *FLT3* WT (Figs. 1*E* and S1*A*) (22, 23, 24). In three distinct GEP datasets (23, 24, 25), we also observed that AML patients with *FADS1* expression above the median display significantly lower survival rates than those with *FADS1* levels below the median (Fig. 1*F*).

### FADS1 supports AML cell expansion and disease propagation *in vivo*

**Figure 2:**
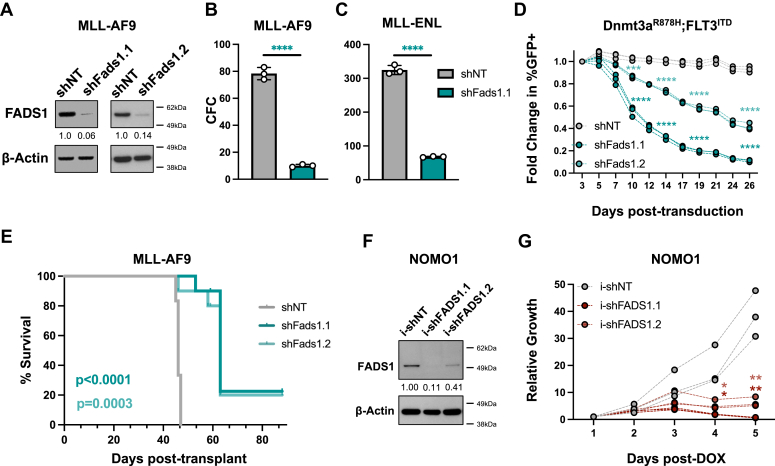
**FADS1 supports AML cell survival and disease propagation *in vivo*.***A*, Western blot analysis of FACS-purified GFP+ MLL-AF9 cells expressing shNT, shFads1.1, or shFads1.2 (*B*) FACS-purified GFP+ MLL-AF9 cells from each shRNA condition were cultured in cytokine-enriched methylcellulose for 7 days (∗∗∗∗*p* < 0.0001). *C*, FACS-purified MLL-ENL cells from each shRNA condition were cultured in cytokine-enriched methylcellulose for 7 days (∗∗∗∗*p* < 0.0001). *D*, growth curve of Dnmt3a^R878H^; FLT3^ITD^-expressing mouse AML cells depicting fold change in % GFP+ cells over time (∗∗∗∗*p* < 0.0001 for all time points except day 7, shNT *versus* shFads1.2, ∗∗∗*p* = 0.0003). *E*, overall survival of mice transplanted with shNT, shFads1.1, or shFads1.2-expressing MLL-AF9 cells. Log-rank (Mantel-cox) test (n = 6 per group). *F*, Western blot analysis of FACS-purified GFP+ NOMO1 cells expressing shNT, i-shFADS1.1, or i-shFADS1.2, 48 h post-2 μg/ml doxycycline (DOX) treatment. *G*, cells from each inducible shRNA condition were treated with 2 μg/ml DOX and then counted at the indicated time points using flow cytometry counting beads on a LSRII flow cytometer (i-shNT *versus* i-shFADS1.1: ∗*p* = 0.0153, day 4 and ∗∗*p* = 0.0015, day 5; i-shNT *versus* i-shFADS1.1: ∗*p* = 0.0351, day 4 and ∗∗*p* = 0.0029, day 5). *Dots* represent individual data points and error bars represent SD. AML, acute myeloid leukemia; ENL, eleven nineteen protein; FACS, fluorescence-activated cell sorting; FADS1, fatty acid desaturase 1; *MLL*, mixed lineage leuekmia; shFADS1, shRNAs that reduce Fads1 protein expression; shNT, nontargeting control shRNA.

We next investigated whether FADS1 has a functional role in AML cell biology. Having observed that FADS1 expression was elevated in *MLL*-rearranged AML, we first performed loss-of-function analyses using shRNA-mediated knockdown in mouse models of AML, driven either by MLL-AF9 or MLL-eleven nineteen protein (ENL) (20, 21, 22). Briefly, mouse AML cells–expressing MLL-AF9 or MLL-ENL were transduced with recombinant lentiviruses that coexpress GFP and either nontargeting control shRNAs (shNT) or mouse shRNAs that reduce FADS1 protein expression (shFads1.1 or shFads1.2) (Fig. 2*A*). shRNA-expressing cells were purified by fluorescence-activated cell sorting and then plated in cytokine-enriched methylcellulose to assess colony-forming capacity (CFC). Depletion of Fads1 protein significantly reduced the CFC of MLL-AF9 and MLL-ENL cells (Figs. 2, *B* and *C*, and S1*B*). Since FADS1 was also elevated in AMLs bearing FLT3^ITD^, we also assessed how shRNA-mediated inhibition impacted the expansion of a mouse AML cell line expressing FLT3^ITD^ and a Dnmt3a^R878H^ mutation (homologous to the DNMT3A^R882H^ mutant found in human AML). Fads1 inhibition significantly impeded the expansion of Dnmt3a^R878H^;FLT3^ITD^ cells, as well as MLL-AF9 cells, in liquid culture (Figs. 2*D* and S1*C*). Given that AA is a Fads1 product, we evaluated whether AA supplementation would impact the antileukemia effects of Fads1 inhibition. The addition of 20 μM AA partially restored the CFC of MLL-AF9 expressing Fads1-targeting shRNAs in semisolid culture (Fig. S1*D*).

We next assessed how Fads1 inhibition impacted the ability of MLL-AF9 cells to induce leukemia in syngeneic mice. Recipients transplanted with MLL-AF9 leukemia cells expressing shFads1.1 (*p* < 0.0001) or shFads1.2 (*p* = 0.003) displayed significantly longer latencies of disease onset than control animals (Fig. 2*E*). Flow cytometric analysis of bone marrow (BM) samples from the mice that developed frank leukemia in each shRNA condition showed that all recipients of shNT-expressing leukemia cells displayed high percentages of GFP+ cells (Fig. S1*E*). However, many of the recipient mice transplanted with shFads1.1- or shFads1.2-expressing leukemia cells displayed significantly lower percentages of GFP+ (*i.e.*, shRNA-expressing) cells (Fig. S1*E*).

We also employed a doxycycline-inducible shRNA approach to assess how inhibition of FADS1 expression impacted the growth of human AML cells. Similar to our observations in mouse leukemia cells, inducible expression of FADS1-targeting shRNAs (i-shFADS1.1 or i-shFADS1.2) impeded the growth of human AML cell lines expressing MLL-AF9 (NOMO1 and MOLM14) as well as that of a normal-karyotype AMLcell line, OCI-AML3 (Figs. 2, *F* and *G*, and S1, *F*–*I*).

### FADS1-targeting shRNAs drive leukemia cell cycle arrest, maturation, and death

**Figure 3:**
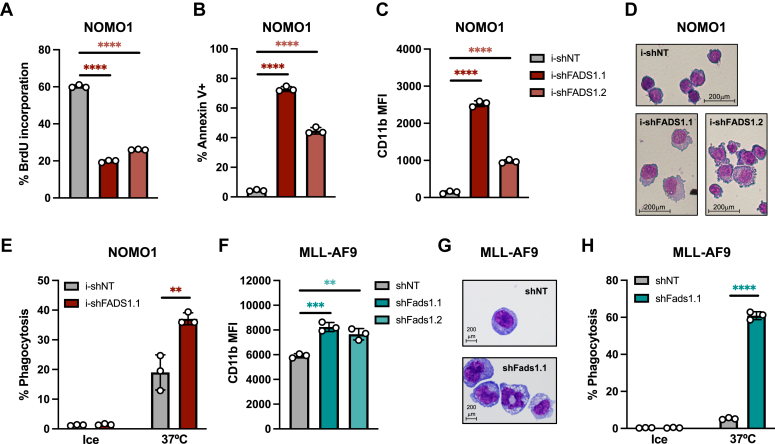
**FADS1 disruption promotes cell cycle arrest, maturation, and death.***A*–*D*, NOMO1 cells expressing i-shNT, i-shFADS1.1, or i-shFADS1.2 were treated with 2 μg/ml DOX treatment and then assessed for the following: (*A*) BrdU-incorporation at 48 h post-DOX (bar plots represent % of cells in S phase); (*B*) % annexin-V+ by flow cytometry at 4 days post-DOX; (*C*) CD11b median fluorescence intensity (MFI) by flow cytometry at 4 days post-DOX; and (*D*) Wright-Giemsa staining at 5 days post doxycycline induction (40× magnification) (∗∗∗∗*p* < 0.0001). *E*, percent of internalized fluorescently labeled (PE) *Escherichia coli* peptides by NOMO1 cells expressing i-shNT, i-shFADS1.1 at 72 h post doxycycline induction. *F*, GFP+ MLL-AF9 cells expressing shNT, shFads1.1, or shFads1.2 were analyzed for CD11b MFI by flow cytometry on day 5 posttransduction (shNT *versus* shFads1.1, ∗∗∗*p* = 0.005 and shNT *versus* shFads1.2, ∗∗*p* = 0.0034). *G*, Wright-Giemsa staining of MLL-AF9 cells expressing the indicated shRNAs 8 days posttransduction. (100× magnification). *H*, percent of internalized fluorescently labeled *E. coli* peptides by MLL-AF9 cells expressing shNT or shFads1.1 as measured by flow cytometry at day 9 posttransduction. Data represent the mean ± SD of three replicates (∗∗∗∗*p* < 0.0001). *Dots* represent individual data points and error bars represent SD. BrdU, bromodeoxyuridine; DOX, doxycycline; FADS1, fatty acid desaturase 1; *MLL*, mixed lineage leuekmia; shNT, nontargeting control shRNA.

We next investigated the consequences of FADS1 inhibition on AML cell biology. FADS1-targeting shRNAs significantly reduced the ability of NOMO1 cells to incorporate the nucleotide analog bromodeoxyuridine compared to controls (Fig. 3*A*), suggesting that FADS1 inhibition reduced the proliferative capacity of NOMO1 cells. Furthermore, FADS1 inhibition led to increased cell death as measured by annexin V positivity, though FADS1 inhibition was less effective in inducing the death of OCI-AML3 than NOMO1 and MOLM14 (Figs. 3*B*, and S2, *A* and *B*). We also observed that FADS1 knockdown increased the expression of the mature myeloid cell marker CD11b on NOMO1, MOLM14, and OCI-AML3 cells (Figs. 3*C*, and S2, *C* and *D*). We next carried out Wright-Giemsa staining to assess how FADS1 inhibition impacted AML cell morphology. NOMO1 and MOLM14 cells expressing FADS1-targeting shRNAs were more asymmetric and displayed fragmented edges as well as a greater cytoplasm-to-nucleus ratio than control cells (Figs. 3*D* and S2*E*). We then assessed the impact of FADS1 inhibition on the phagocytic capacity of AML cells using *Escherichia coli* peptides fused to a pH-sensitive red dye (pHrodo Red, Thermo Fisher Scientific) that only fluoresces in acidic environments such as that of the phagolysosome. Inhibition of FADS1 increased the ability of NOMO1 cells to engulf these *E. coli* particles (Figs. 3*E* and S2*F*). Mouse AML cells expressing Fads1-targeting shRNAs also displayed increased CD11b expression and phagocytic capacity as well as similar morphological features to those observed in human cells (Figs. 3, *F*–*H*, and S2, *G* and *H*). These results indicate that AML cells rely on FADS1 to properly cycle, survive, and maintain an immature state as well as plasma membrane morphology.

### Disruption of PUFA biosynthesis causes lipidome remodeling and depletes 20:4- FAs from major lipid classes

**Figure 4:**
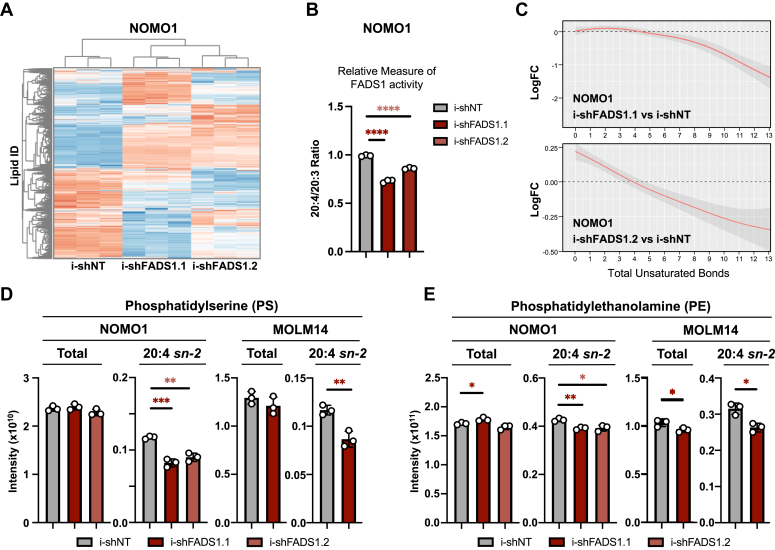
**FADS1 inhibition selectively depletes 20:4 fatty acid in phospholipids.***A*, hierarchical clustering heatmap of MS-based untargeted lipidomics analysis of NOMO1 cells expressing i-shNT, i-shFADS1.1, or i-shFADS1.2 at 40 h post doxycycline induction (n = 3 replicates per group). *B*, ratios of total signals from phospholipids containing 20:4 or 20:3 FA in the sn-2 position (∗∗∗∗*p* < 0.0001). *C*, analysis of fatty acid composition for i-shFADS1 *versus* control i-shNT cells based on the total MS signal (lipid abundance) with the specified total degree of unsaturation. *Top panel*, represents the comparison between i-shFADS1.1 *versus* control i-shNT and the *bottom panel* represents the comparison between i-shFADS1.2 *versus* control i-shNT. *D* and *E*, both total and 20:4, *sn-2* levels of (*D*) phosphatidylserine (PS) (NOMO1 –i-shNT *versus* i-shFADS1.1: 20:4 *sn-2* PS, ∗∗∗*p* = 0.0005; i-shNT *versus* i-shFADS1.2: 20:4 *sn-2* PS, ∗∗*p* = 0.0015; and MOLM14 – i-shNT *versus* i-shFADS1.1: 20:4 *sn-2* PS, ∗∗*p* = 0.0059) and (*E*) phosphatidylethanolamine (PE) were determined by MS-based untargeted NOMO1 cells expressing i-shNT, i-shFADS1.1, or i-shFADS1.2 and MOLM14 cells expressing i-shNT or i-shFADS1.1 at 40 h, post-DOX treatment (NOMO1 – i-shNT *versus* i-shFADS1.1: total PE, ∗*p* = 0.0284; and 20:4 *sn-2* PE, ∗∗*p* = 0.0023; and i-shNT *versus* i-shFADS1.2: 20:4 *sn-2* PE, ∗*p* = 0.015; MOLM14 – i-shNT *versus* i-shFADS1.1, 20:4 total PE, ∗*p* = 0.045; i-shNT *versus* i-shFADS1.1, 20:4 *sn-2* PE, ∗*p* = 0.0107). *Dots* represent individual data points and error bars represent SD. FADS1, fatty acid desaturase 1; shFADS1, shRNAs that reduce Fads1 protein expression; shNT, nontargeting control shRNA.

To assess the impact of FADS1 depletion on AML lipid composition, we extracted complex lipids from control and shFADS1-expressing NOMO1 and MOLM14 cells and performed global untargeted lipidomics (Fig. 4*A*). As a proxy for FADS1 enzyme activity, we calculated the ratio of all phospholipid species containing the FAs 20:4 (including the FADS1 product, AA) to those containing 20:3 FAs (including the FADS1 substrate, dihomo-gamma-linolenic acid in both shRNA conditions. From this analysis, we observed that the ratio of 20:4 to 20:3 species was significantly lower in shFADS1-expressing NOMO1 and MOLM14 cells than control cells (Figs. 4*B* and S3*A*). However, it should be noted that the double bond position within a FA cannot be discerned by the methodology used in this study, and therefore it is possible that the 20:4/20:3 ratio could be skewed by non-AA 20:4– or non–dihomo-gamma-linolenic acid 20:3–containing FA species. Consistent with the desaturase function of FADS1, the saturation levels of lipid species overall were increased in both NOMO1 and MOLM14 cells upon FADS1 depletion (Figs. 4*C* and S3*B*). Specifically, we observed decreased incorporation of 20:4 FAs into the membrane phospholipids, phosphatidylserine (PS) (Fig. 4*D*) and phosphatidylethanolamine (PE) (Fig. 4*E*), though total PS and PE levels were largely unaffected by FADS1 inhibition. We also found that FADS1 inhibition led to decreased incorporation of 20:4 FAs into storage lipids, cholesteryl esters (Fig. S3*C*), and triglycerides (TGs) (Fig. S3, *D* and *E*, middle panels). FADS1 inhibition also resulted in a significant decrease in the incorporation of 20:5 FAs, including the 20:5 FA eicosapentaenoic acid (the product of FADS1 desaturation of the ω-3 FA, eicosatetraenoic acid), into TGs (Fig. S3, *D* and *E*, right panels).

### Proinflammatory signaling cascades accompany disruption in PUFA biosynthesis

**Figure 5:**
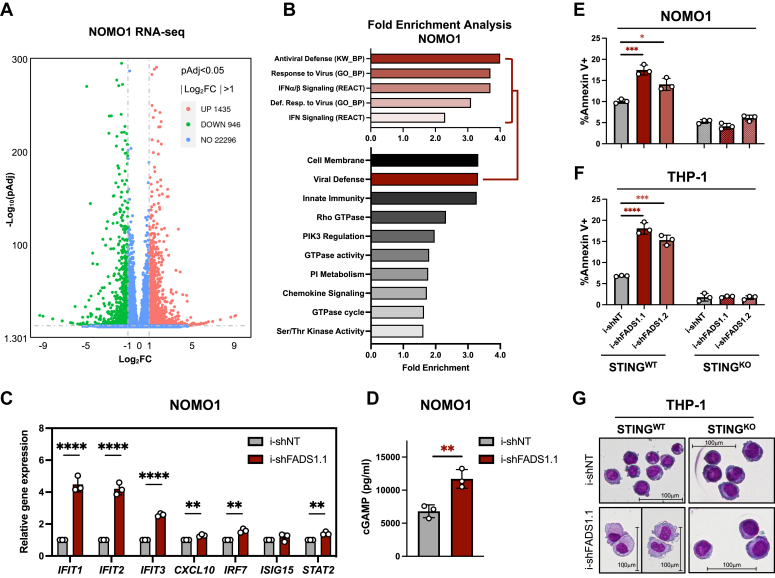
**FADS1 inhibition drives STING-mediated AML cell maturation and death.***A*, *volcano plot* of differentially expressed genes from a RNA-seq analysis of NOMO1 cells expressing i-shNT or i-shFADS1.1 and treated with DOX for 40 h. *B*, *bottom panel*, pathway enrichment analysis using the DAVID functional annotation tool and included GO_CC, GO_BP, GO_MF, UP_KW biological processes, KEGG, and reactome pathways. *Top panel*, pathway enrichment breakdown of “Viral Defense” cluster as depicted in the *bottom panel*. *C*, quantitative polymerase chain reaction analysis of the specified genes in i-shNT-, i-shFADS1.1-expressing NOMO1 cells at 40 h post doxycycline induction (i-shNT *versus* i-shFADS1.1: *IFIT1*, ∗∗∗*p* = 0.0002; *IFIT2*, ∗∗∗*p* = 0.0001; *IFIT3*, ∗∗∗∗*p* < 0.0001; *CXCL10*, ∗∗0.0024; *IRF7*, ∗∗*p* = 0.0012; *STAT2*, ∗∗*p* = 0.0025). *D*, cytoplasmic extracts from NOMO1-expressing i-shNT or i-shFADS1.1 and treated with DOX 40 h earlier were subjected to ELISA to detect cGAMP levels (*p* = 0.0078). *E*, NOMO1-STING^WT^ (*i.e.*, WT). and NOMO1-STING^KO^ cells expressing i-shNT, i-shFADS1.1, or i-shFADS1.2 were analyzed for % annexin V^+^ (i-shNT *versus* i-shFADS1.1, ∗∗∗*p* = 0.006; i-shNT *versus* i-shFADS1.2, ∗*p* = 0.0104) using flow cytometry 4 days post-DOX induction. *F*, THP-1-STING^WT^ (*i.e.*, WT). and THP-1-STING^KO^ cells expressing i-shNT, i-shFADS1.1, or i-shFADS1.2 were analyzed for % annexin V^+^ (i-shNT *versus* i-shFADS1.1, ∗∗∗*p* = 0.0001; i-shNT *versus* i-shFADS1.2, ∗∗∗*p* = 0.0002) using flow cytometry 5 days post-DOX induction. *G*, Wright-Giemsa staining of THP-1-STING^WT^ and THP-1-STING^KO^ cells expressing i-shNT or i-shFADS1.1, or i-shFADS1.2 shRNA at 5 days post doxycycline induction (20× magnification). *Dots* represent individual data points and error bars represent SD. AML, acute myeloid leukemia; BM, bone marrow; cGAMP, 2′3′-cGMP-AMP; FADS1, fatty acid desaturase 1; shNT, nontargeting control shRNA; STING, stimulator of interferon genes.

To gain further insight into the downstream molecular consequences of FADS1 inhibition, we performed RNA-Seq of NOMO1 cells expressing i-shNT or i-shFADS1.1. After filtering all differentially expressed genes for an adjusted *p* value of 0.05 (Benjamini–Hochberg false discovery rate) and a fold change of 1, we identified 1435 upregulated and 946 downregulated differentially expressed genes in shFADS1 cells compared to shNT controls (Fig. 5*A*). We next used the functional annotation tool, DAVID (26), to perform a pathway enrichment analysis on genes that were differentially regulated in shFADS1 by a factor of at least 2. This analysis revealed that many molecular pathways associated with inflammation, in particular type-1 interferon signaling driven by STING, were enriched upon FADS1 inhibition (Figs. 5*B* and S4*A*). These results were validated by quantitative polymerase chain reaction (qPCR) for select interferon-stimulated genes (Figs. 5*C* and S4*B*). We also observed that FADS1 knockdown leads to the accumulation of 2′3′-cGMP-AMP (cGAMP), an endogenous activator of STING (Figs. 5*D* and S4*C*). Using gene set enrichment analysis (GSEA) (27, 28), we also observed that molecular signatures related to toll-like receptor (TLR) signaling (*e.g.*, TLR1, TLR4, and TLR6) were enriched in FADS1-depleted cells (Fig. S4*D*). Western blot analysis revealed that protein levels of TLR1 were increased following FADS1 inhibition, however, we were unable to detect TLR4 or TLR6 (Fig. S4*E* and data not shown).

### STING ablation mitigates the antileukemia effects of disrupting PUFA biosynthesis

We next investigated whether the activation of STING signaling is related to the antileukemia effects mediated by disruption of PUFA biosynthesis. STING is an ER-localized protein that, once activated, initiates type-1 interferon transcriptional programs and in some cases cell death (16). Since FADS1 knockdown activated STING-mediated interferon signaling in AML cells and that STING agonists have shown antileukemia potential (14, 16), we assessed how CRISPR/Cas9-mediated deletion of STING (STING^KO^; Fig. S4*F*) impacted the antileukemia effects of FADS1 inhibition. While shRNA-mediated inhibition of FADS1 promoted cell death in control (STING^WT^) NOMO1 and THP-1 cells, these phenotypes were blunted in isogenic cell lines lacking STING (Fig. 5, *E* and *F*). Moreover, STING^KO^ mitigated the maturation induced by FADS1-targeting shRNAs in THP-1 cells by blunting both the increase in CD11b levels and the morphological changes (Figs. 5*G* and S4*G*). We also observed that STING deletion attenuated the induction of several interferon stimulated genes that are induced by FADS1 inhibition (Fig. S4*H*).

### Pharmacological inhibitors of FADS enzymes exhibit anti-AML activity

**Figure 6:**
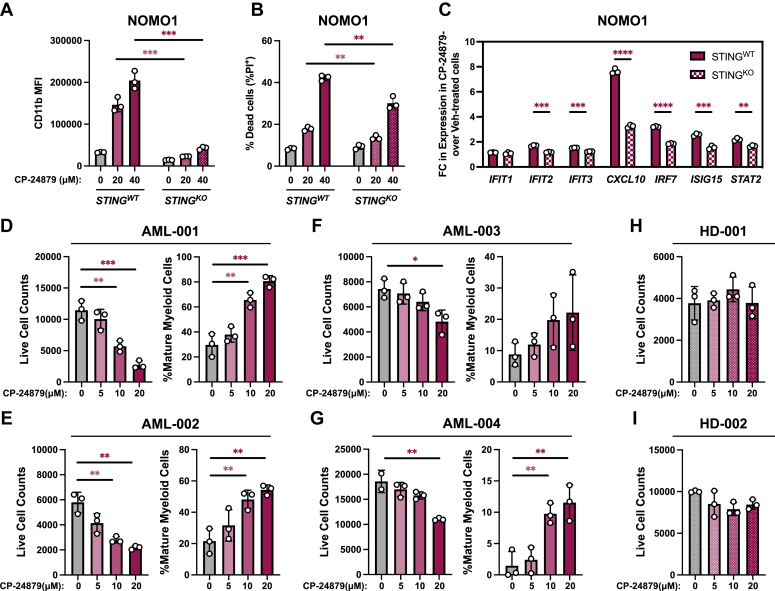
**Pharmacological inhibition of FADS enzymes imparts anti-AML activity.***A* and *B*, NOMO1 STING^WT^ and STING^KO^ were treated with the indicated concentrations of CP-24879 for 48 h and then assessed by flow cytometry for the following: *A*, CD11b MFI (∗∗∗*p* = 0.0002 for both of the indicated comparisons) and (*B*) % annexin V^+^ (20 μM STING^WT^*versus* 20 μM STING^KO^, *p* = 0.0074, and 40 μM STING^WT^*versus* 40 μM STING^KO^, *p* = 0.0029). *C*, complimentary DNA recovered from NOMO1-STING^WT^ and NOMO1-STING^KO^ cells treated with vehicle or 40 μM CP-24879 were analyzed by quantitative polymerase chain reaction for the expression of the indicated genes. The data is presented as the fold change (FC) in gene expression of CP-24879–treated cells over vehicle-treated cells in each cellular genotype (STING^WT^*versus* STING^KO^: *IFIT2*, ∗∗∗*p* = 0.0002; *IFIT3*, ∗∗∗*p* = 0.0001; *CXCL10*, ∗∗∗∗*p* < 0.0001; *IRF7*, ∗∗∗∗*p* < 0.0001; *ISIG15*, ∗∗∗*p* = 0.0005; *STAT2*, ∗∗*p* = 0.0023). *D*–*G*, BM or peripheral blood (PB) samples recovered from patients diagnosed with AML were treated with the indicated concentrations of CP-24879 for 4 days and then analyzed by flow cytometry to count live AML cells (*left panels*, hCD45^Low^, hCD14^Low^, hCD16^Low^, P.I.^−^) and the percent of mature myeloid cells (*right panels*, hCD45^+^, hCD14^High^, hCD16^High^, P.I.^−^). *D*, AML patient #1: live cell counts, 0 *versus* 10 μM, ∗∗*p* = 0.0053 and 0 *versus* THP-1- 20 μM, ∗∗∗*p* = 0.0009; and mature myeloid cells, 0 *versus* 10 μM, ∗∗*p* = 0.0047 and 0 *versus* 20 μM, ∗∗∗*p* = 0.001. *E*, AML patient #2: live cell counts, 0 *versus* 10 μM, ∗∗*p* = 0.0037 and 0 *versus* 20 μM, ∗∗*p* = 0.0016; and mature myeloid cells, 0 *versus* 10 μM, ∗∗*p* = 0.0099 and 0 *versus* 20 μM, ∗∗*p* = 0.0028. *F*, AML patient #3: live cell counts, 0 *versus* 20 μM, ∗*p* = 0.0199. *G*, AML patient #4: live cell counts, 0 *versus* 20 μM, ∗∗*p* = 0.0079; and mature myeloid cells, 0 *versus* 10 μM, ∗∗*p* = 0.0067 and 0 *versus* 20 μM, ∗∗*p* = 0.0086. *H* and *I*, BM samples recovered from healthy donors (*H*) #1 and (*I*) #2 were treated with the indicated concentrations of CP-24879 for 4 days and then analyzed by flow cytometry to count live cells (hCD45^+^, P.I.^−^). *Dots* represent individual data points and error bars represent SD. AML, acute myeloid leukemia; BM, bone marrow; FADS1, fatty acid desaturase 1; P.I., propidium iodide; STING, stimulator of interferon genes.

We next investigated whether pharmacologic disruption of PUFA biosynthesis impacted AML biology. In the absence of a proven selective FADS1 inhibitor, we utilized the small molecule CP-24879, which is dual inhibitor of FADS1 and FADS2 (29). Increasing doses of CP-24879 selectively impaired the CFC of MLL-AF9–expressing mouse AML cells but not that of normal mouse hematopoietic stem and progenitor cells (Fig. S5*A*). Furthermore, CP-24879 treatment induced a dose-dependent increase in CD11b expression and annexin-V positivity in both NOMO1 and THP-1 AML cell lines (Fig. S5, *B* and *C*). NOMO1 and THP-1 cells treated with CP-24879 also underwent morphological changes resembling myeloid maturation (Fig. S5, *B* and *C*, right panels). This induction of CD11b expression and cell death mediated by CP-24879 treatment was significantly diminished by STING deletion (Figs. 6, *A* and *B*, and S5*D*). Similar to FADS1 knockdown, CP-24879 treatment also induced interferon-stimulated genes, which was attenuated by deletion of STING (Figs. 6*C* and S5*E*).

Since CP-24879 inhibits the activity of both FADS1 and FADS2, we sought to investigate the contribution of each enzyme to the antileukemia effects of CP-24879 by comparing it to a FADS2-specific inhibitor, sc-26196. Increasing concentrations of both CP-24879 and sc-26196 induced dose-dependent cell death of NOMO1 and MOLM14 cells, though CP-24879 was more effective than sc-26196 at killing NOMO1 cells at 40 μM (Fig. S5, *F* and *G*). In THP-1 cells, only CP-24879 induced cell death in a dose-dependent manner (Fig. S5*H*). While CP-24879 treatment robustly induced CD11b expression in a dose-dependent manner, sc-26196 failed to induce the expression of CD11b on NOMO1, MOLM14, or THP-1 cells, even at higher concentrations (40 μM) (Fig. S5, *I*–*K*).

To ensure that the antileukemia activity of CP-24879 was not restricted to cultured human and mouse AML cells, we evaluated the impact of CP-24879 treatment on nine PD-AML samples and one sample from a patient diagnosed with myelodysplastic syndrome (Table S2). All of the nine patient samples that were treated with 20 μM CP-24879 displayed a significant reduction in live cell count, though the margin of decrease compared to vehicle-treated cells varied from sample to sample (Figs. 6, *D*–*G*, and S6, *A*–*F*). In comparison to vehicle-treatment, six patient samples displayed significantly lower cells numbers when treated with 10 μM CP-24879 (Figs. 6, *D* and *E*, and S6, *A*, *C*, *D*, and *F*), whereas live cell counts were significantly decreased by 5 μM CP-24879 in only one sample (AML-006) (Fig. S6*B*). We also measured how CP-24879 treatment impacted the maturation state of four patient samples. From this analysis, we found that 10 μM and 20 μM CP-24879 significantly increased the percentage of mature myeloid cells in three patients (Figs. 6, *D*, *E*, and *G*), whereas the fourth displayed an increase in mature myeloid cells that failed to reach significance (Fig. 6*F*). We also assessed how CP-24879 impacted the viability of BM-derived hematopoietic cells from healthy donors. In contrast to PD-AML, the number of live cells from three separate healthy donors was not significantly altered by treatment of increasing concentrations of CP-24879 (Figs. 6, *H* and *I* and S6*G*).

### STING agonism potently eliminates AML cells but also reduces healthy granulocytes

**Figure 7:**
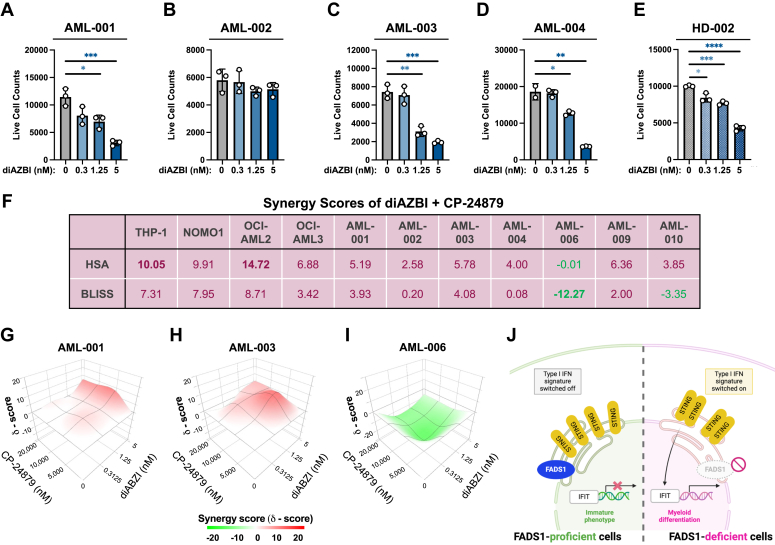
**STING agonism effectively eliminates patient-derived AML cells and variably cooperates with FADS1 inhibition.***A*–*D*, BM or PB samples recovered from patients diagnosed with AML were treated with the indicated concentrations of diAZBI for 4 days and then analyzed by flow cytometry to count live AML cells. *A*, AML-001: live cell counts, 0 *versus* 10 μM, ∗*p* = 0.0171 and 0 *versus* 20 μM, ∗∗∗*p* = 0.0009. *B*, AML-002: no significant differences. *C*, AML-003: live cell counts, 0 *versus* 10 μM, ∗∗*p* = 0.0014 and 0 *versus* 20 μM, ∗∗∗*p* = 0.0003. *D*, AML-004: live cell counts, 0 *versus* 10 μM, ∗*p* = 0.0189 and 0 *versus* 20 μM, ∗∗*p* = 0.0011. *E*, BM samples recovered from HD-002 were treated with the indicated concentrations of diAZBI for 4 days and then analyzed by flow cytometry to count live CD45+ cells. 0 nM *versus* 0.312 nM, ∗*p* = 0.0101; 0 nM *versus* 1.25 nM, ∗∗∗*p* = 0.0001; 0 nM *versus* 5 nM, ∗∗∗∗*p* < 0.0001). *Dots* represent individual data points and error bars represent SD. *F*, tabulation of the HSA (*middle row*) or BLISS (*bottom row*) synergy scores for the combination of diAZBI and CP-24879 assessed in the indicated cell lines or patient-derived AML samples. *G*–*I*, three dimensional plots of the synergy/cooperation scores (*y*-coordinate) for the indicated patient-derived AML samples treated with varying combinations of the STING agonist, diAZBI (*z*-coordinate), and CP-24879 (*x*-coordinate). *J*, a graphical summary of the molecular consequences of disrupting PUFA biosynthesis in AML. AML, acute myeloid leukemia; BM, bone marrow; FADS1, fatty acid desaturase 1; PB, peripheral blood; PUFA, polyunsaturated fatty acid; STING, stimulator of interferon genes.

Based on our observation that disruption of PUFA biosynthesis leads to STING activation and subsequent AML cell death, we next investigated whether STING activation was sufficient to phenocopy FADS1 inhibition on AML biology, using a STING agonist, diAZBI (30). To initially test the effectiveness and specificity of diAZBI, we treated THP-1-STING^WT^ and THP-1-STING^KO^ cells with increasing concentrations of diAZBI from 1 to 100 nM. While 10 and 100 nM were highly effective in eliminating THP-1-STING^WT^ cells, THP-1-STING^KO^ cells were largely unaffected by diAZBI treatment (Fig. S7*A*). Having confirmed the specificity of diAZBI as a STING agonist, we next tested its activity on malignant and healthy human hematopoietic cells. diAZBI was effective in eliminating nine of ten malignant patient samples, with the only exception being AML-002, which is derived from a patient with myelodysplastic syndrome (Figs.7, *A*–*D* and S7, *B*–*G*). Having observed that concentrations between 0.1 to 10 nM diAZBI displayed varying antileukemia potential, we next evaluated this concentration range on BM cells derived from healthy donor samples. Similar to our observations in PD-AML samples, diAZBI significantly decreased live cell numbers in two of the three healthy donor samples in a dose-dependent manner (Figs. 7*E* and S7*H*). This reduction in overall healthy BM cell counts was largely due to a decline in granulocytes as diAZBI treatment did not significantly lower lymphocyte numbers (Fig. S7, *I* and *J*).

### Combined targeting of FADS enzymes and STING displays varying cooperativity in AML cell lines and PD-AML

Given the antileukemia properties of CP-24879 and STING agonists, we next investigated whether these compounds cooperate in eliminating AML cells. We first tested this combination on the AML cell lines, THP-1, NOMO1, OCI-AML2, and OCI-AML3 cells and found that diAZBI and CP-24879 display additive cooperativity (31) in reducing the viability of all four cell lines (Figs. 7*F* and S8, *A*–*D*). We next performed synergy analyses on seven of the ten patient samples that we obtained. Using two separate synergy scoring models (HSA and BLISS), we observed that CP-24879 and diABZI positively cooperated in five of the seven patient samples tested (Figs. 7, *F*–*I* and S9, *A* and *B*). While CP-24879 and diAZBI displayed an antagonistic relationship on AML-006, the two drugs were determined to have both a cooperative and an antagonistic relationship on AML-010, depending on the statistical model applied (Figs 7, *F* and *I* and S9*A*). Having observed that diAZBI reduced the live cell counts of healthy BM cells and in particular granulocytes, we next compared the live cell counts of patient and healthy donor samples treated with vehicle or varying combinations of diAZBI and CP-24879 to identify a potential therapeutic window. The addition of CP-24879 did not exacerbate the effects of diAZBI on total healthy donor cells (Fig. S10*A*). As single treatments or in combination, 0.3125 nM diAZBI and either 5 μM or 10 μM CP-24879 minimally impacted PD-AML live cell counts in comparison with healthy donor BM cells (Fig. S10, *B*–*F*). Relative to vehicle treatment, 1.25 nM diAZBI, alone or in combination with 5 μM or 10 μM CP-24879 displayed a greater decline in live cell counts in three PD-AML samples compared to healthy donors (Fig. S10, *G*–*I*). Lastly, 5 nM diAZBI, alone or in combination with 5 μM or 10 μM CP-24879, displayed a greater fold reduction in live cell counts for four PD-AML samples compared to healthy BM cells (Fig. S10, *J*–*L*). Collectively, these data suggest that disruptions in PUFA biosynthesis mediated by FADS inhibition leads to lipidomic remodeling and STING-mediated maturation and destruction of AML cells (Fig. 7*J*).

## Discussion

PUFAs display pleiotropic functions in animal cell physiology and have been implicated in a variety of diseases (*e.g.*, cancer), especially those of metabolic origin (32, 33). Here, we report that certain aggressive subtypes of AML are highly dependent on the PUFA biosynthetic enzyme, FADS1. Previous work has shown that FADS1 supports laryngeal squamous cell carcinoma progression (9), while reduced Δ5 desaturase may predict poorer outcomes in non–small cell lung cancer (34). Despite these seemingly contrasting observations, a recent report that mined The Cancer Genome Atlas data to investigate the association between FADS1 mRNA levels and survival outcomes in over 11,000 subjects (distributed across numerous cancer types) found that patients with higher FADS1 have worse disease-free survival and overall survival rates (35). From a retrospective analysis of GEPs from AML patients and healthy donors, we show that patient AML samples display increased levels of *FADS1* mRNA compared to healthy donors and that elevated FADS1 expression correlates with worse outcomes. These findings parallel those of Pabst *et al.* that reported that AML patients, particularly those that are high risk, have increased levels of plasma AA, a product of FADS1 ω-6 desaturation (12). AML can arise from a multitude of distinct genetic abnormalities (as well as combinations thereof) and is therefore classified into multiple subtypes with varying outcomes. We have observed that *FADS1* expression is elevated in complex-karyotype AML, normal-karyotype-AML as well as AMLs bearing *MLL* rearrangements or FLT3^ITD^. We have also seen that inhibition of FADS1 antagonizes AML cell growth and survival in experimental models of *MLL*-rearranged– or FLT3^ITD^-AML indicating that FADS1 plays an important functional role in the pathogenesis of these AML subtypes. In line with these data, we also found that two (AML-005 and AML-007) of the three PD-AML samples that were most sensitive to the FADS1/2 inhibitor, CP-24879 carried alterations in their *FLT3* gene. However, further studies in a larger cohort of patient AML samples will be required to determine which AML genetic subtypes are reliant on FADS1.

At the molecular level, we have demonstrated that FADS1 inhibition causes AML cells to undergo lipid remodeling with significant changes in the composition of membrane lipids. Upon FADS1 inhibition, overall saturation levels increase and 20:4 FAs are depleted from certain phospholipids (*e.g.*, PS and PE) and neutral lipids (*e.g.*, TG and cholesteryl ester). We also observed some cell type–specific differences in certain lipid species. For example, NOMO1, but not MOLM14 cells, displayed a significant decrease in 20:4, *sn-2*–containing phosphatidylglycerols, total Hex2 ceramides (hexosyl ceramide with two hexoses), and total sphingomyelins upon FADS1 inhibition (not shown). Furthermore, while FADS1 inhibition resulted in increased levels of total phosphatidic acids and cardiolipins in NOMO1 cells, we observed the opposite trend in MOLM14 cells (not shown). This difference could be due to the distinct genetic makeup of each cell line as MOLM14 cells bear a FLT3^ITD^, whereas NOMO1 do not. However, an assessment of a larger sample size (and possibly isogenic experimental models) will be needed to address these differences.

In addition to disruption of phospholipids, FADS1 knockdown caused a significant upregulation of inflammatory pathways, including STING and TLR signaling. STING activation has been shown to induce interferon-dependent cell death in human AML cells (16), and TLR stimulation can lead to growth arrest and terminal differentiation of leukemic blasts (36, 37). We have found that STING ablation mitigated the increased maturation and cell death phenotypes induced by FADS1 inhibition. While our data do not demonstrate a cause-and-effect relationship between the disruption in PUFA biology mediated by FADS1 inhibition and STING activation in AML, several previously reported correlative observations suggest that this may be a possibility. First, our lipidomic analyses showed that the concentration of certain MUFAs such as palmitoleic acid and oleic acid decreases across various lipid classes upon FADS1 knockdown (Fig. S11*A*), which might be due to lower stearoyl-coenzyme A desaturase 1 expression (Fig. S11, *B* and *C*). MUFA alterations have been shown to drive STING-dependent type-1 interferon signaling in T cells (38). Second, we have found that shRNA-mediated inhibition of FADS1 also indirectly leads to significantly lower *FADS2* transcript levels (Fig. S11, *B* and *C*). Others have shown that FADS2 binds to and inhibits STING, thus establishing a negative feedback loop since STING itself can diminish FA desaturation (39). Third, Barnett *et al.*, previously reported that the phosphatidylinositol PI(4,5)P2 is responsible for tethering c-GAS to the plasma membrane and that disrupting PI(4,5)P2 levels can activate c-GAS and subsequent STING activation (40). While we did not observe a significant difference in PI(4,5)P2 in our lipidomic datasets, the imbalances in the PUFA content mediated by inhibition of FADS enzymes could promote membrane stiffening, which in turn, could (although not previously reported) influence the activation state of membrane bound proteins such as STING and c-GAS. However, future studies will be required to establish whether alterations in PUFA biosynthesis are directly or indirectly linked to STING activation.

Impaired interferon signaling is a common mechanism of immune dysfunction in cancer (41). Indeed, we found that several AML cell lines as well as pediatric AML patients carry interferon pathway mutations and deep deletions (Table S3 and Fig. S12), suggesting that dysregulation interferon-associated pathways might contribute to leukemogenesis. Our data also indicate the activation of interferon signaling *via* STING agonism has cell autonomous potency in AML. Two recent studies indicate that STING agonism could also attenuate AML growth and survival through nonautonomous cellular mechanisms (16, 42), suggesting that targeting STING in AML may have multiple antileukemia effects by simultaneously targeting leukemia cells as well as their tumor-supportive microenvironment.

From a therapeutic perspective, we did observe that the dual small molecule inhibitor of FADS1 and FADS2, CP-24879 displayed varying antileukemia activity on PD-AML samples without significantly harming healthy BM-derived hematopoietic cells. Similar to CP-24879, the STING agonist displayed antileukemia activity, however, diAZBI was also effective in eliminating healthy granulocytes. While we observed strong antileukemia cooperativity between CP-24879 and diABZI in cultured human AML cell lines, the cooperative relationship varied among PD-AML samples. Since PUFA biosynthesis and STING signaling play important roles in other tissues outside of the hematopoietic system, additional *in vivo* studies using animal models of AML will be needed to assess the safety and effectiveness of targeting FADS enzymes and/or STING. Interestingly, CP-24879 and diAZBI are each well tolerated at a dose of 3 mg/kg in mice (29, 30). The effectiveness and safety of these small molecules could also be improved with drug delivery systems such as lipid nanoparticles, as has been seen for diAZBI in mouse models of breast cancer (43, 44). Altogether, our results uncover a previously unrecognized dependency for PUFA biosynthesis in AML and unveil a new potential therapeutic strategy.

## Experimental procedures

### Cell culture and lentiviral infections

All leukemia cell lines were obtained either from the American Type Culture Collection or the German Collection of Microorganism and Cell Cultures (DMSZ). Human cell lines were cultured in RPMI medium supplemented with 10% tetracycline-depleted fetal bovine serum and penicillin/streptomycin, while the murine cell lines MLL-AF9 and MLL-ENL were cultured in the above-described RPMI medium supplemented with 10 ng/ml murine stem cell factor (Peprotech), 6 ng/ml mIL-6 (Peprotech), and 5 ng/ml mIL-3 (Peprotech). All cell lines were authenticated by IDEXX BioAnalytics and periodically monitored in-house to ensure that they were mycoplasma-free using Hoechst staining. shRNA constructs targeting human FADS1, and control vector (nontargeting or shNT) were purchased from Horizon (shFADS1.1 – *Horizon ID V3SH11252-227897479* (Mature antisense: TAGGCATCTAGCCAGAGCT) and shFADS1.2 – *Horizon ID V3SH11252-228281995* (Mature antisense: GGGAATATGGTTCATCTGT). pLentiCRISPRv2-STING_gRNA3 was a gift from Nicolas Manel (Addgene plasmid # 127640; http://n2t.net/addgene:127640; RRID: Addgene 127640). plentiCRISPRv2 was a gift from Feng Zhang (Addgene plasmid # 52961; http://n2t.net/addgene:52961; RRID: Addgene 52,961). The shRNA and CRISPR/Cas9 constructs, packaging, and envelope plasmids—psPAX and pMD, respectively—were transfected into HEK293TL cells using X-tremeGENE (Sigma-Aldrich) 9 to produce high-titer lentiviral media as previously described. The human leukemia cell lines NOMO1 and MOLM-14 were transduced with shRNA-expressing recombinant lentiviruses plus polybrene (8 μg/ml). Stable cell lines were generated *via* puromycin selection (2 μg/ml for 72 h immediately after transduction then maintained in culture at 0.5 μg/ml). Human monocytes THP-1-STING^WT^ and THP-1-STING^KO^ were purchased from Invivogen.

### Mouse studies

All animal studies were approved by the Fox Chase Cancer Center Institutional Animal Care and Use Committee, protocol #13-01. shRNA constructs targeting mouse Fads1 and control vector (nontargeting or shNT) were cloned into the pLKO vector (shFads1.1: TAGGCATCTAGCCAGAGCT and shFADS1.2: CCGGAATGTGGACTGGGTC). The shRNA constructs, packaging, and envelope plasmids—psPAX and pMD, respectively—were transfected into HEK293TL cells using X-tremeGENE (Sigma-Aldrich) 9 to produce high-titer lentiviral media. For *in vivo* survival assays, we utilized a mouse model driven by the human MLL-AF9 fusion gene as described previously (45, 46). Briefly, BM cells recovered from leukemic mice were spin-infected with lentiviruses (multiplicity-of-infection 0.4–0.6) supplemented with polybrene (8 mg/ml) in 12-well nonadherent plates (2200 rpm at 30 °C for 90 min). Forty-eight hours after transduction, 150000 GFP+ cells were sorted at 48 h posttransduction and then transplanted into sublethally irradiated (450 rad) syngeneic recipient male mice. Experimental groups were randomized based on age (8–12 weeks).

### Patient and healthy donor samples

Human sample collection was approved by the Washington University Institutional Review Board, protocol #201011766. All human studies were conducted in accordance with the “Declaration of Helsinki” principles. Deidentified samples were cultured in RPMI-1640 supplemented with 10% FBS, 1% Penicillin/Streptomycin, 1% GlutaMax, 1% nonessential amino acids, 1% sodium pyruvate, 2% Hepes, 0.9% β-mercaptoethanol, 100 ng/ml human stem cell factor, 10 ng/ml human FMS-like tyrosine kinase 3 ligand, 10 ng/ml human thrombopoietin, 10 ng/ml hIL-3, 20 ng/ml hIL-6, and 50 μg/ml Normocin for 4 to 5 days.

### Lipidomic analysis

The lipidomic analysis was performed at The Wistar Institute Proteomics and Metabolomics Shared Resource on a Thermo Q-Exactive HF-X mass spectrometer, as described previously (47). Methanol-quenched samples with equivalent cell numbers were spiked with 5 μl EquiSPLASH LIPIDOMIX internal standard, and lipids were extracted using a modified Folch extraction (2:1:1 chloroform:methanol: 0.88% sodium chloride). Lipid extracts were dried under nitrogen, resuspended in 100 μl 9:1 methanol:chloroform, and stored at −20 °C before analysis. Samples were analyzed by liquid chromatography (LC)-MS/MS on a Q Exactive HF-X mass spectrometer coupled to a ThermoScientific Vanquish LC System. LC separation was performed by reversed-phase chromatography using a ThermoScientific Accucore C30 LC column. Data were acquired on the mass spectrometer with separate runs for positive and negative polarities. LipidSearch 4.2 (Thermo Fisher Scientific, https://www.thermofisher.com/us/en/home/technical-resources/technical-reference-library/mass-spectrometry-support-center/liquid-chromatography-mass-spectrometry-software-support/lipidsearch-software-support/lipidsearch-software-support-getting-started.html) was used to identify individual lipid species and perform relative quantification based on MS peak area. Detected lipid species were filtered by the expected adduct and identification confidence. Quantification was based on the equivalent percentage of sample injected and was further normalized by EquiSPLASH internal standards for represented lipid classes to account for sample-to-sample differences in extraction efficiency and matrix effect. Data were further normalized to the summed area of identified lipid species. Lipid species displaying significant change (false discovery rate–corrected q-value < 0.05) with at least 1.5-fold change were selected. FA composition analysis (carbon chain length and number of double bonds) was performed using LipidSuite (48). The expression heatmap was generated using the online tool offered by heatmapper (49).

### RNA-seq and qPCR analyses

RNA-seq was performed by Novogene according to the company’s established protocols. Briefly, messenger RNA was purified from total RNA using poly-T oligo-attached magnetic beads. After fragmentation, random hexamer primers were used to synthesize the first strand complimentary DNA, followed by the second strand complimentary DNA synthesis using deoxyuridine triphosphate for directional library. Quantified libraries were pooled and sequenced on an Illumina HiSeq 2500. Raw data (FASTQ files) were processed using Perl scripts. Index of the reference genome was built using Hisat2 v2.0.5 and paired-end reads were aligned to the reference genome using Hisat2 v2.0.5. FeatureCounts v1.5.0-p3 was used to count the reads numbers mapped to each gene. Differential expression analysis was performed using the DESeq2 R package (1.20.0, https://bioconductor.org/packages/release/bioc/html/DESeq2.html). The resulting *p*-values were adjusted using the Benjamin and Hochberg’s approach for controlling the false discovery rate. Genes with an adjusted *p* value ≤0.05 found by DESeq2 were assigned as differentially expressed. Fold enrichment analyses were performed using the DAVID bioinformatics tool (v6.8). GSEA was performed using the GSEA_4.2.3 version Broad Institute). Select targets were confirmed by qPCR (Primers listed in Table S4).

### Cell morphology analysis

A cytocentrifuge (StatSpin Cytofuge, Beckman Coulter) was used to concentrate 30,000 cells in 100 ul volume onto nontreated microscope slides, which were then dried overnight at room temperature. The slides were fixed with 100% methanol for 5 min and after air-drying, and they were stained with 200 μl of Wright-Giemsa (Thermo Fisher Scientific) working solution (6% Wright-Giemsa, 1% acetone in GURR buffer) at room temperature for 15 min. Slides were then rinsed in water and let dry for imaging on a Nikon Eclipse E400.

### Western blot analysis

Total cell lysates were resolved by SDS-PAGE (4%–12% Bis-Tris gels Thermo Fisher Scientific). Proteins were transferred to a polyvinylidene fluoride membrane, and after blocking in 1× tris-buffered saline with 0.1% Tween-20 + 5% nonfat milk for 1 h at room temperature, blots were incubated with primary antibody overnight at 4 °C. Staining with secondary antibody was performed at room temperature for 40 min and blots we developed with Immobilon ECL Ultra Western HRP Substrate (Sigma-Aldrich). The following antibodies were used: Human FADS1 (Abcam, 126706 and Proteintech, 10627-1-AP); Human TLR1 (Cell Signaling, Danvers, 2209); β-Actin (Cell Signaling, 8H10B10). Antibodies were used and validated according to the manufacturer’s instructions. The images of Western blots presented in Figure. 2*D*, and Figs. S1, *F* and *G*, and S4*E* were acquired using exposure of blots to autoradiography film that was then developed using an Kodak X-OMAT developer. Films were then scanned to generate digital images. The digital images of Western Blots presented in Fig. S4*F* were acquired using a Bio-Rad ChemiDoc Imager. Bands were quantified using ImageJ (https://imagej.net/ij/).

### Phagocytosis assay

We used pHrodo Red *E. coli* BioParticles (Thermo Fisher Scientific) to detect phagocytosis *via* flow cytometry. Briefly, cells were incubated with pH-sensitive *E. coli* BioParticles for 15 min at 37 °C or on ice (negative control), as per manufacturer’s instructions. The samples were then analyzed on a BD LSR II Flow Cytometer (Becton Dickinson).

### cGAMP enzyme-linked immunosorbent assay

Cytosolic content was extracted after cell lysis using the ProteoExtract cytosol/mitochondria fractionation kit (QIA88, Sigma-Aldrich). Cell lysates were quantified and 40 μg was used for cGAMP measurement *via* competitive ELISA as per the manufacturer’s instructions (2′3′-cGAMP ELISA Kit, item number 501700, Cayman Chemical).

## Data availability

The datasets generated in the current study are available from the corresponding author upon reasonable request.

## Supporting information

This article contains supporting information (19, 22, 23).
