# Supplementary figures and images for: Disruption of polyunsaturated fatty acid biosynthesis drives STING-dependent acute myeloid leukemia cell maturation and death
Source: J Biol Chem. 2024 Mar 22;300(5):107214. doi: 10.1016/j.jbc.2024.107214 (PMC11061745; doi:10.1016/j.jbc.2024.107214)

# Figure S1

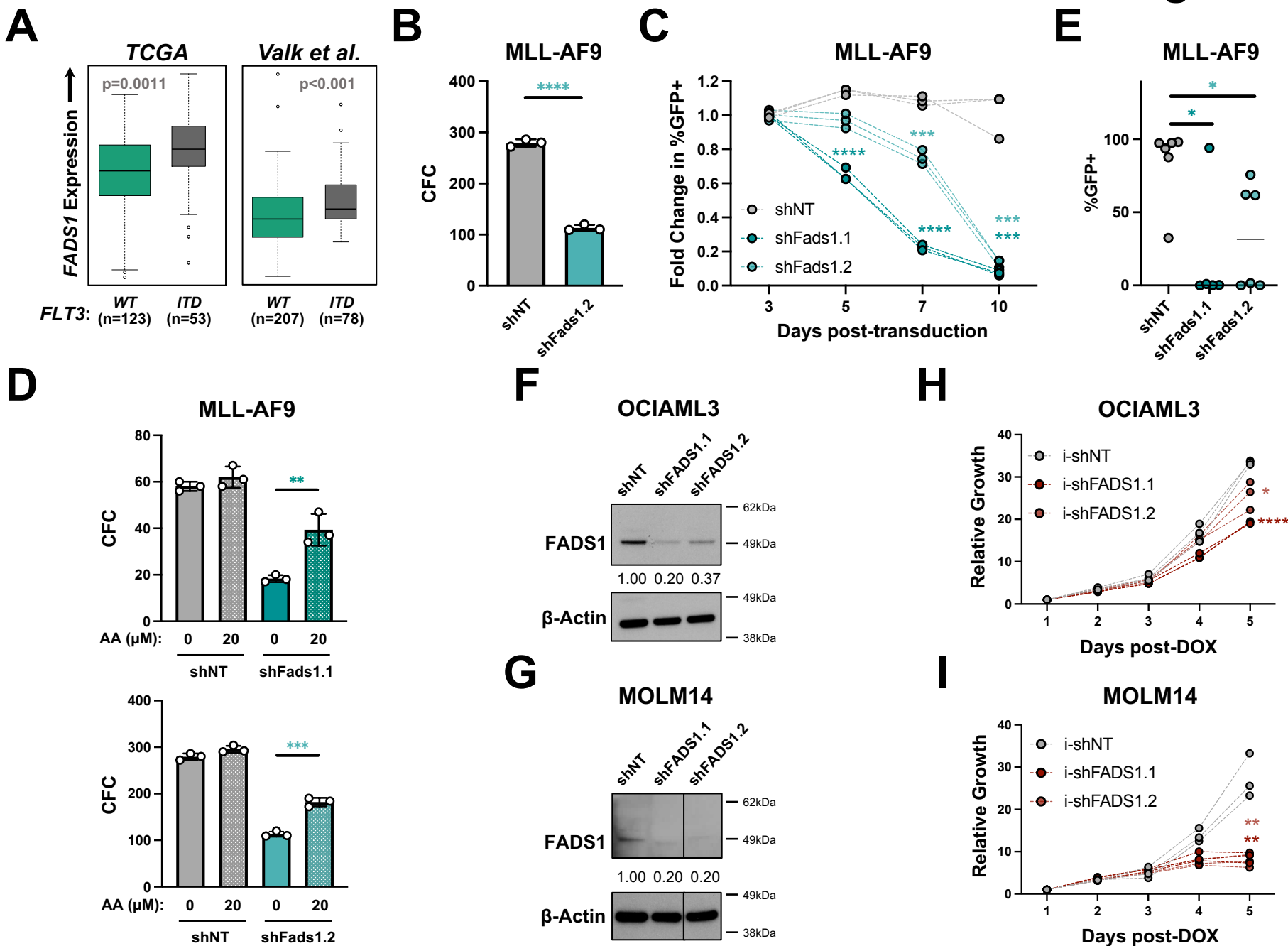

Supplement: Supporting Figure S1 [file mmc1.pdf]

# Figure S2

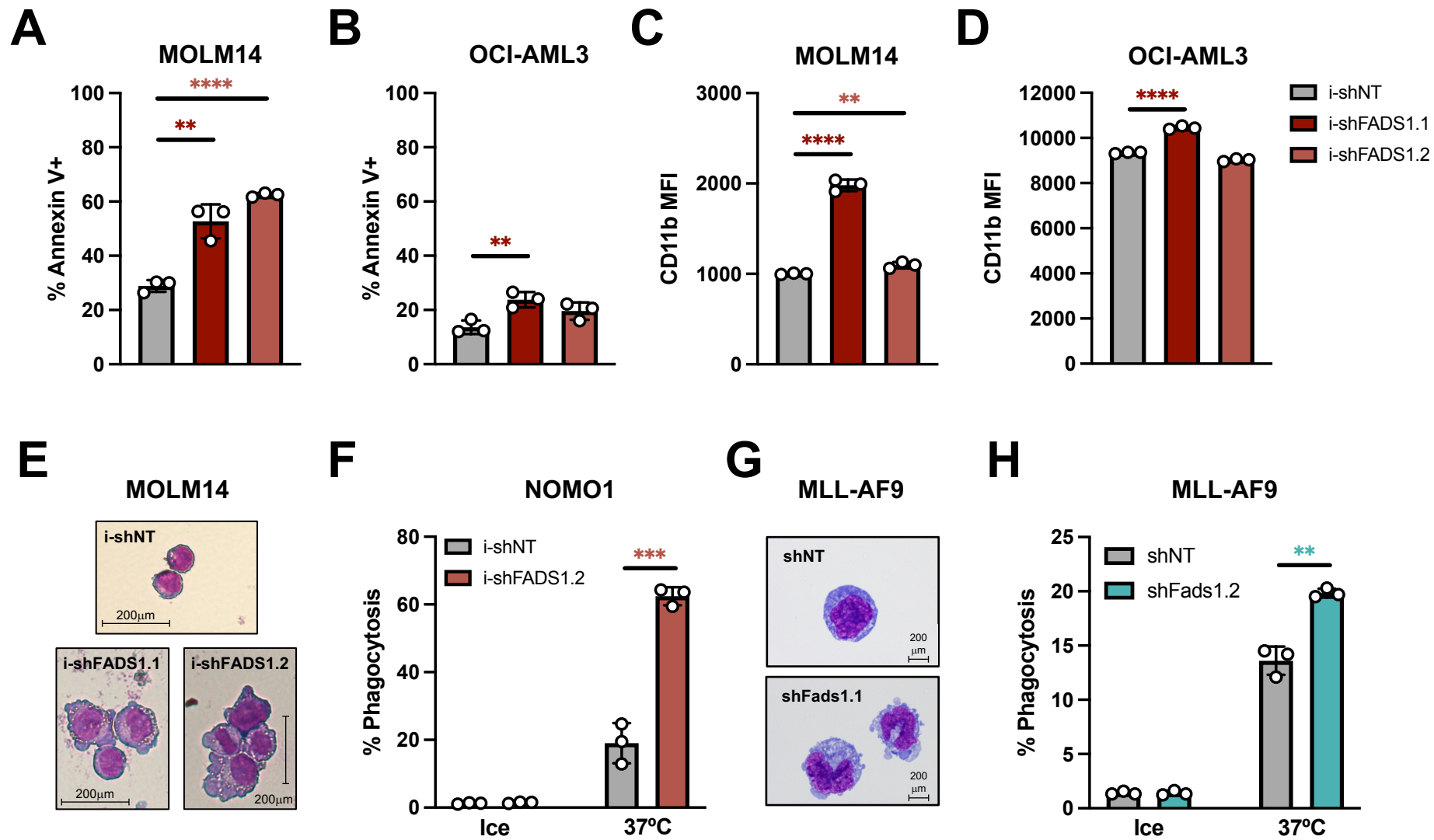

Supplement: Supporting Figure S2 [file mmc2.pdf]

# Figure S3

**A**

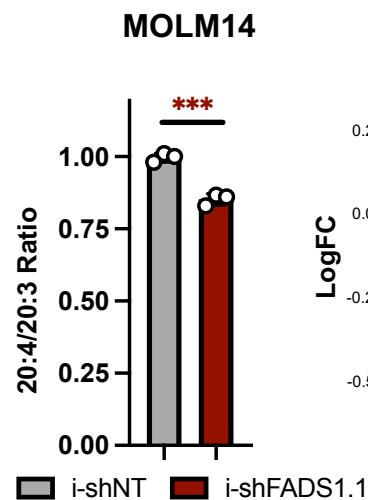

**B**

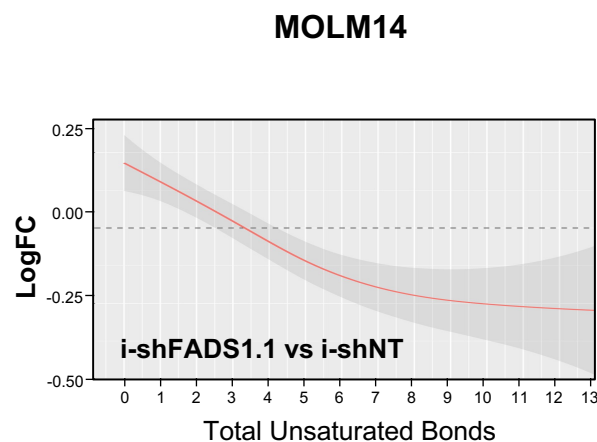

**C**

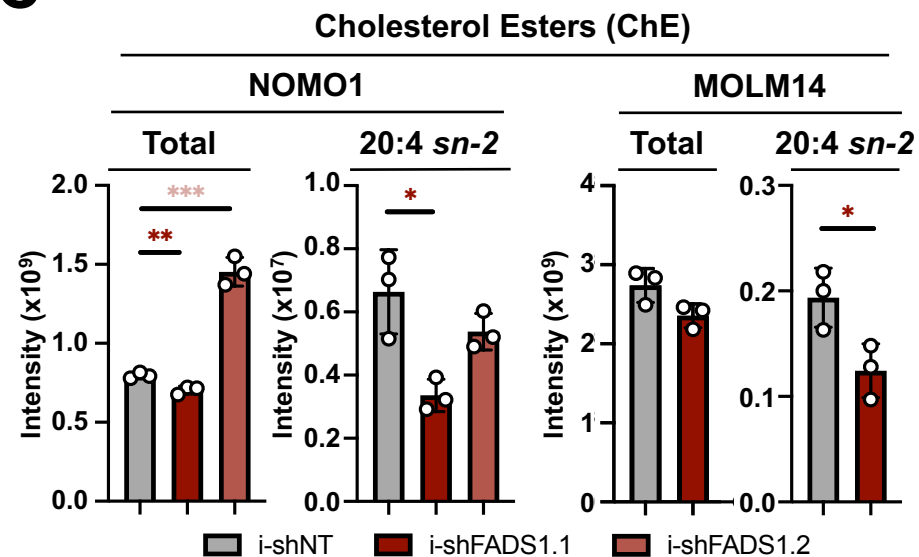

**D**

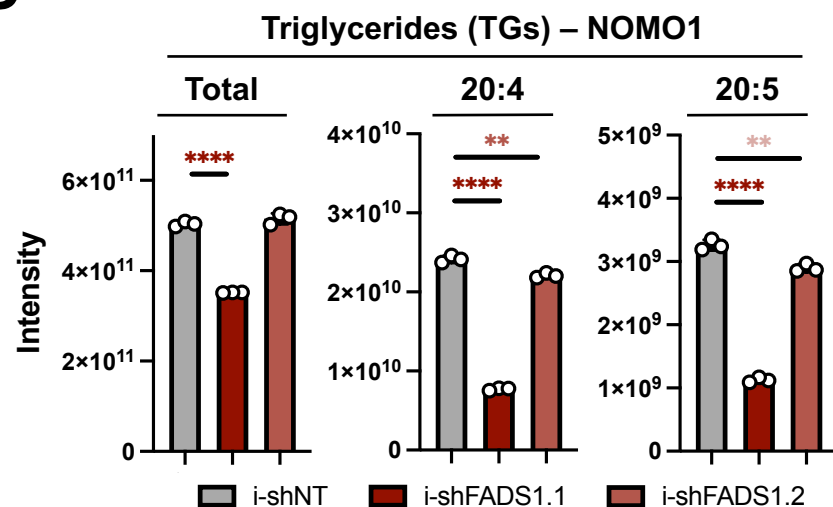

**E**

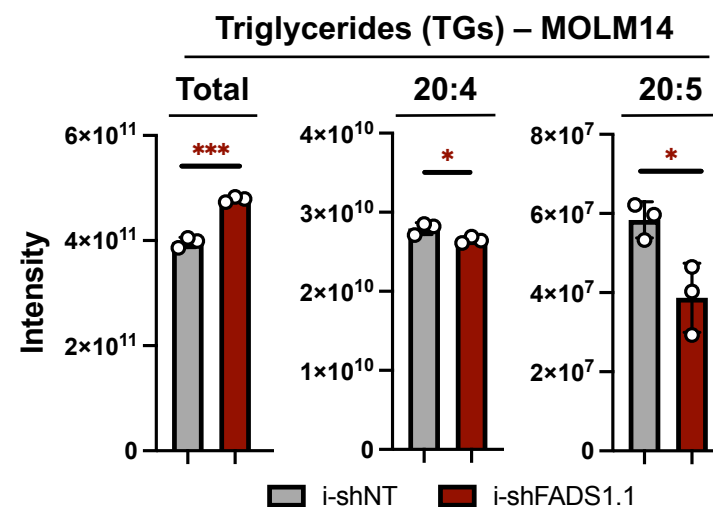

Supplement: Supporting Figure S3 [file mmc3.pdf]

# Figure S4

**A**

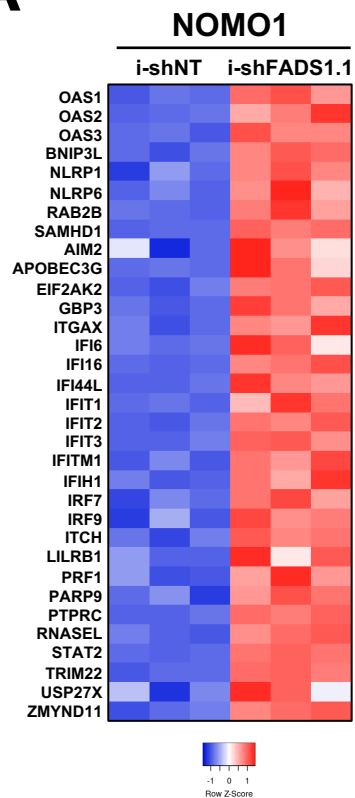

**B**

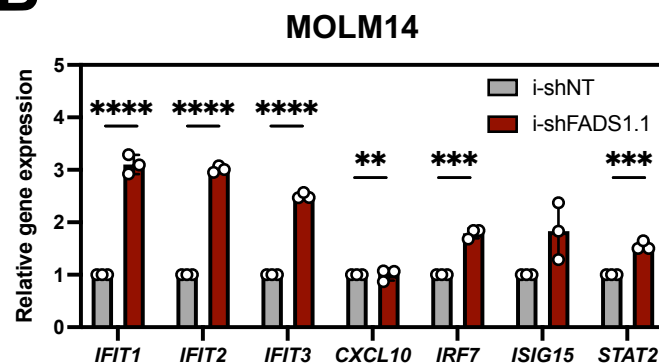

**C**

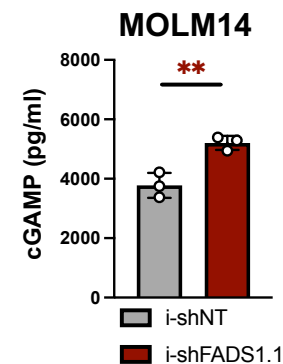

**D**

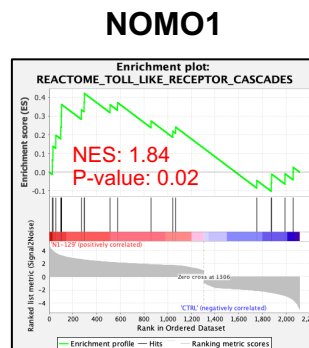

**E**

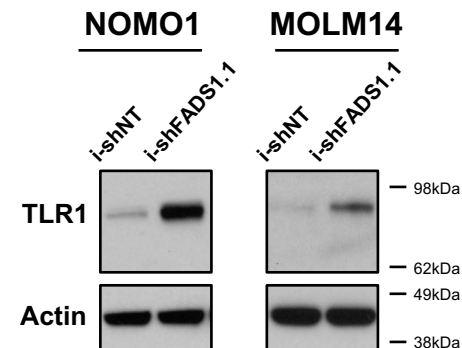

**F**

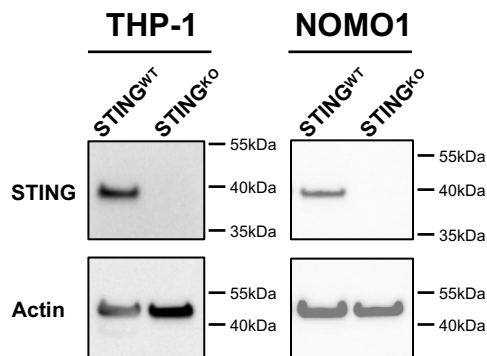

**G**

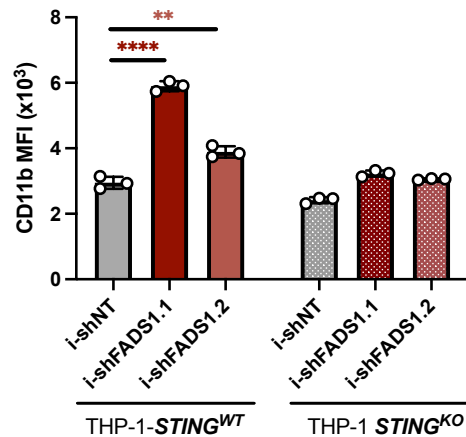

**H**

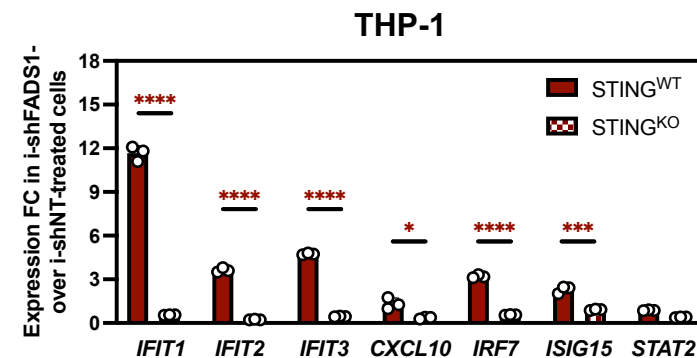

Supplement: Supporting Figure S4 [file mmc4.pdf]

# Figure S5

## A

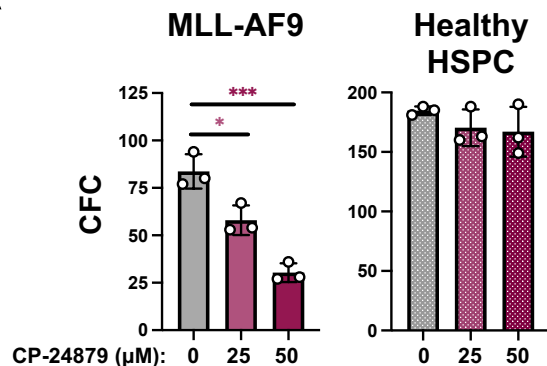

## D

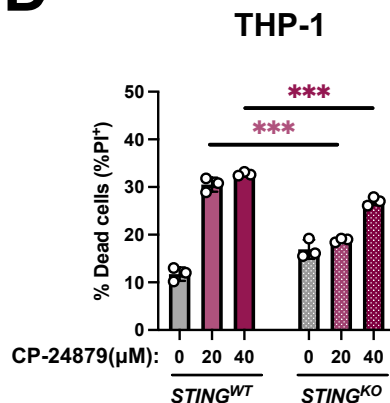

## E

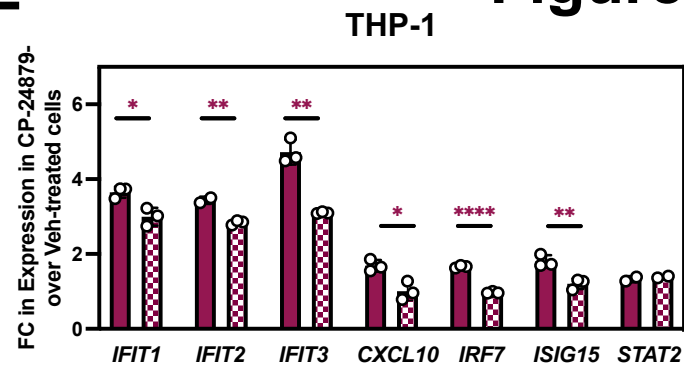

## B

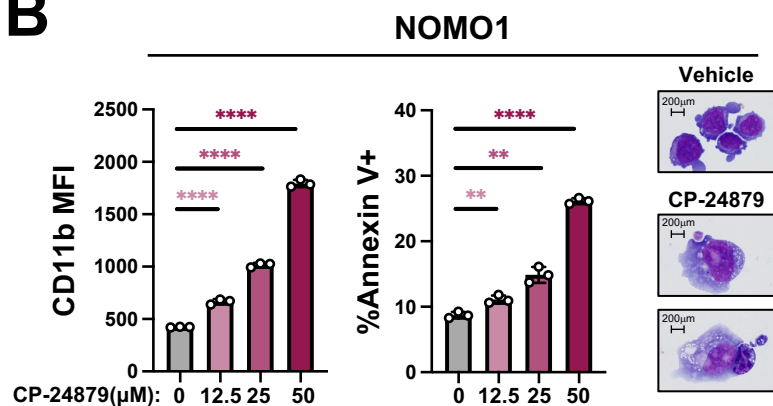

## F

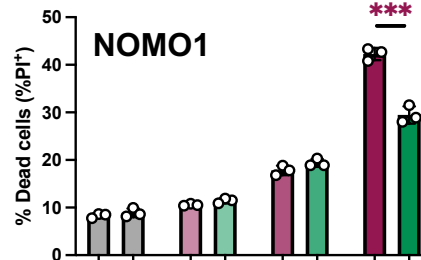

## G

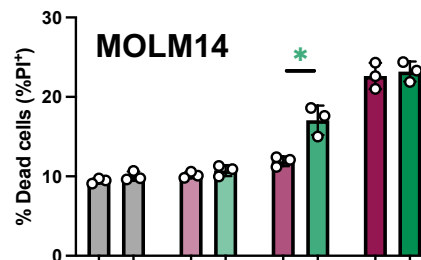

## H

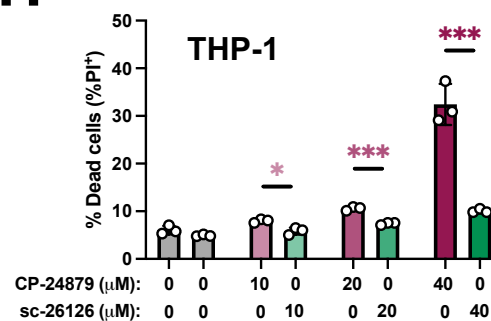

## I

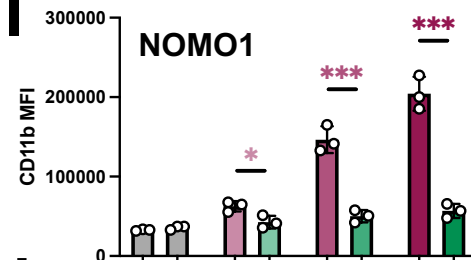

## J

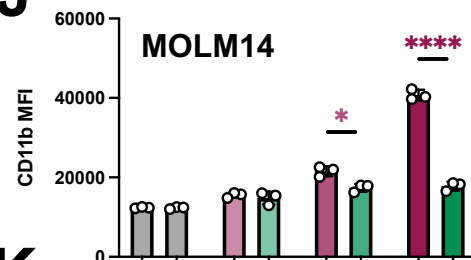

## K

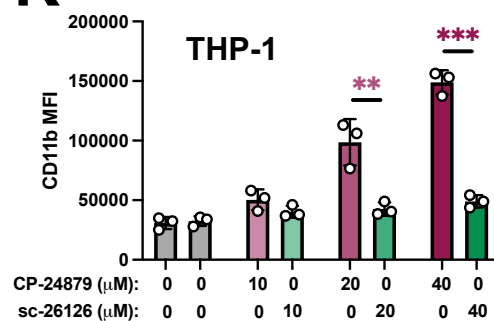

Supplement: Supporting Figure S5 [file mmc5.pdf]

# Figure S6

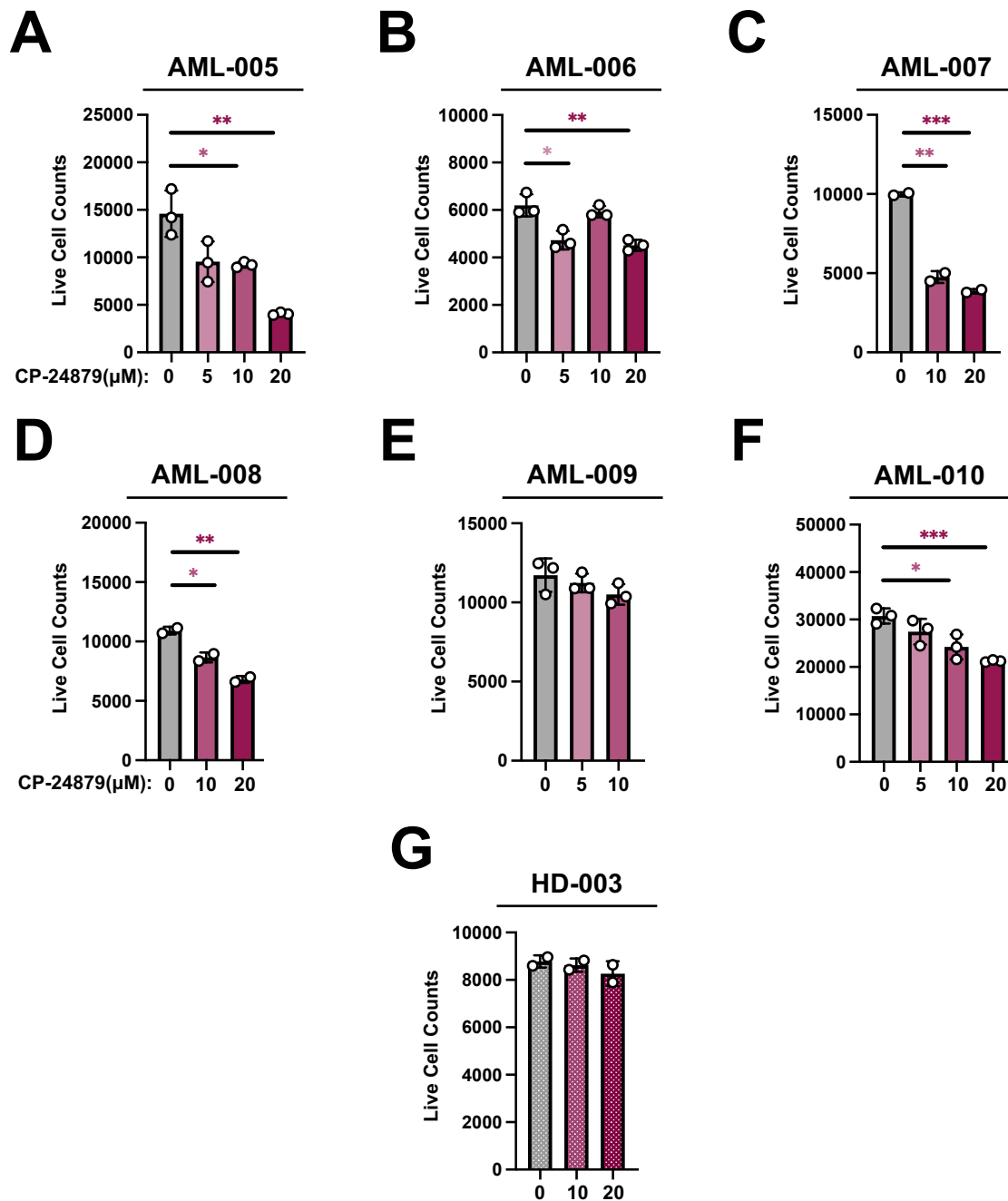

Supplement: Supporting Figure S6 [file mmc6.pdf]

# Figure S7

## A

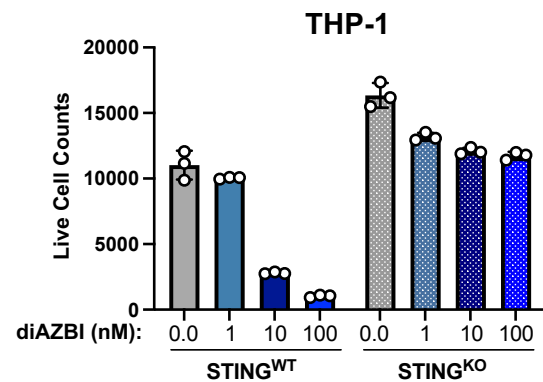

## H

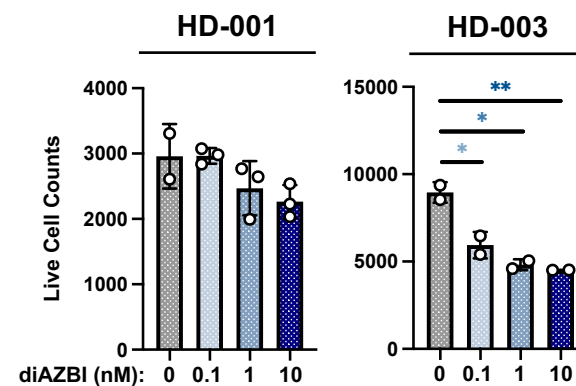

## B

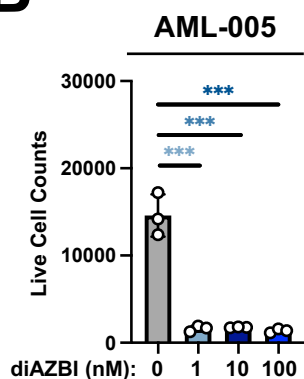

## C

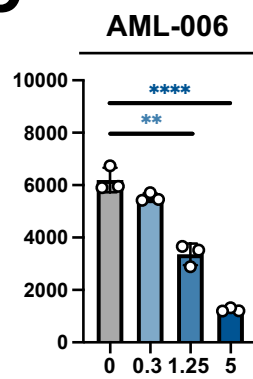

## D

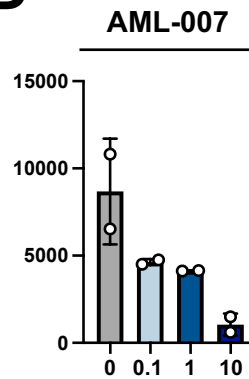

## I

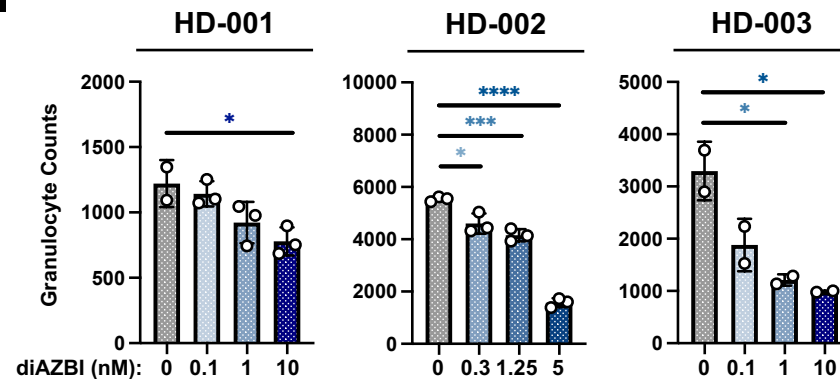

## E

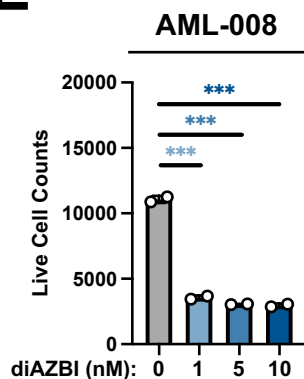

## F

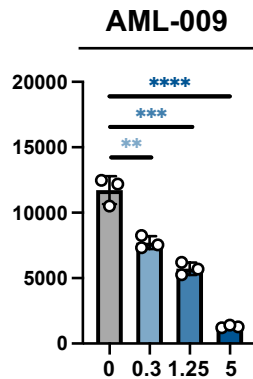

## G

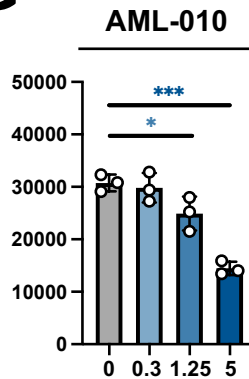

## J

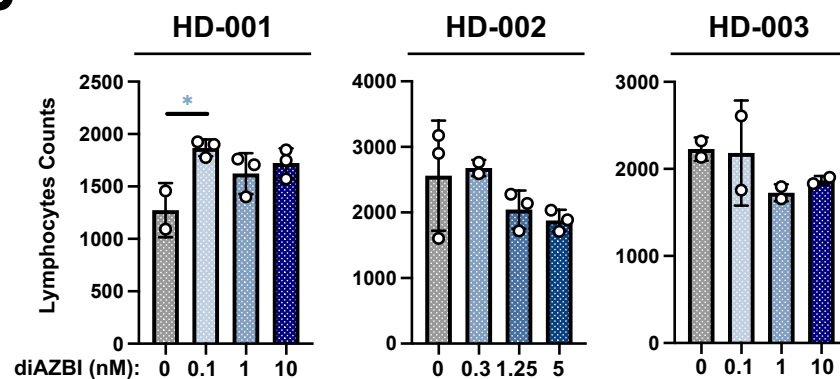

Supplement: Supporting Figure S7 [file mmc7.pdf]

**A**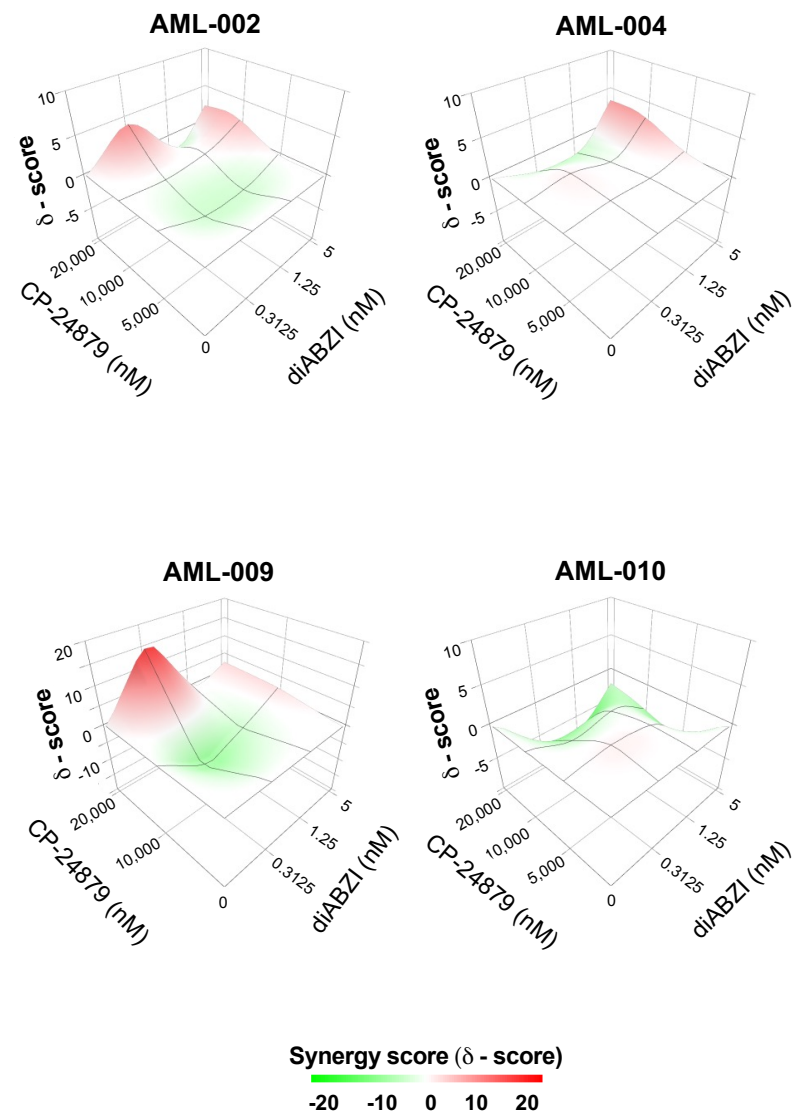**B**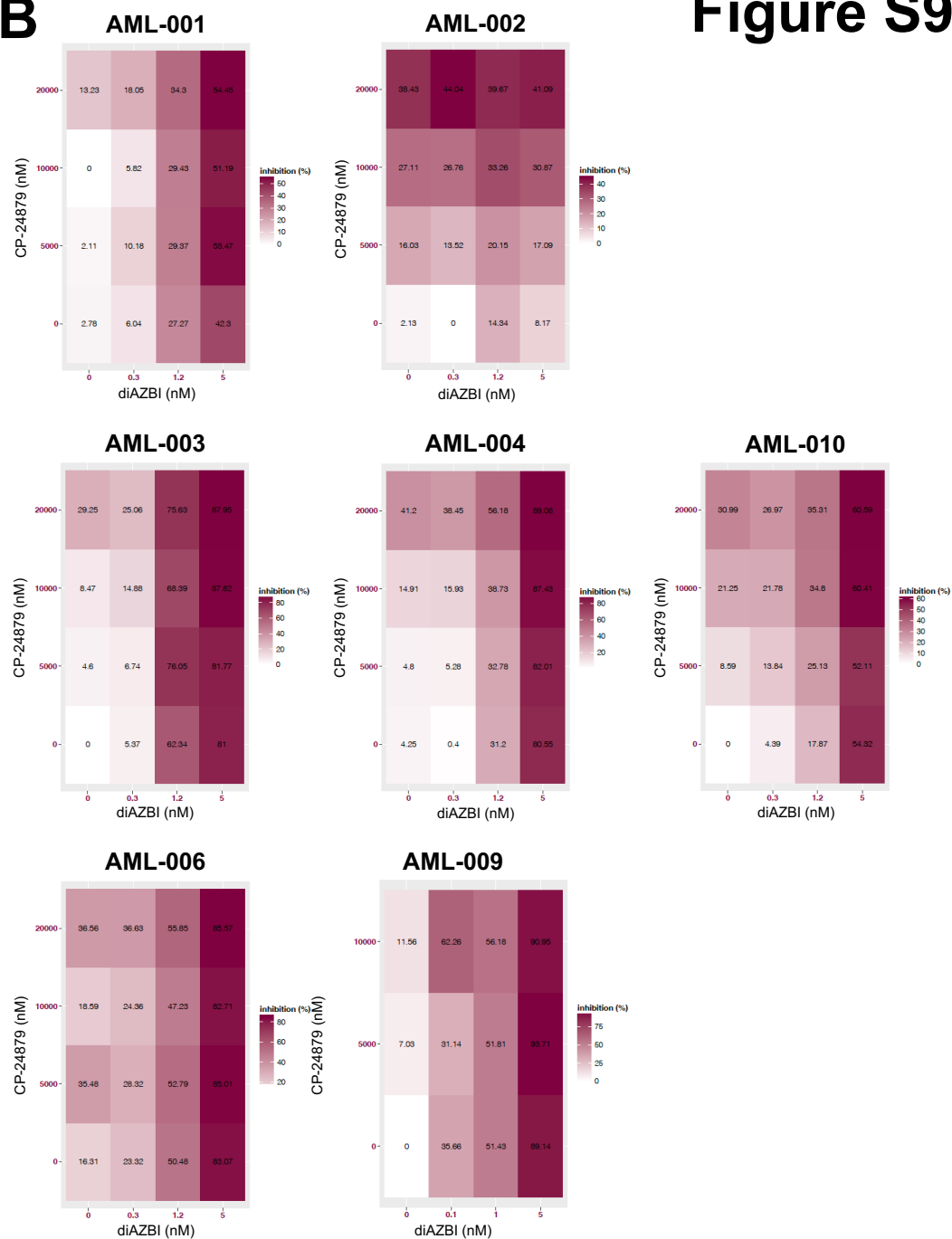**Figure S9**

Supplement: Supporting Figure S9 [file mmc9.pdf]

**A**

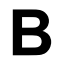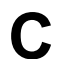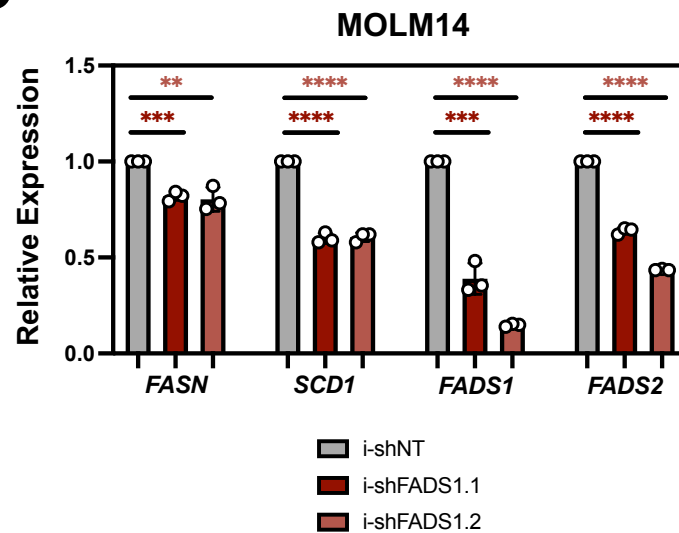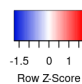

Supplement: Supporting Figure S11 [file mmc11.pdf]

# Figure S12

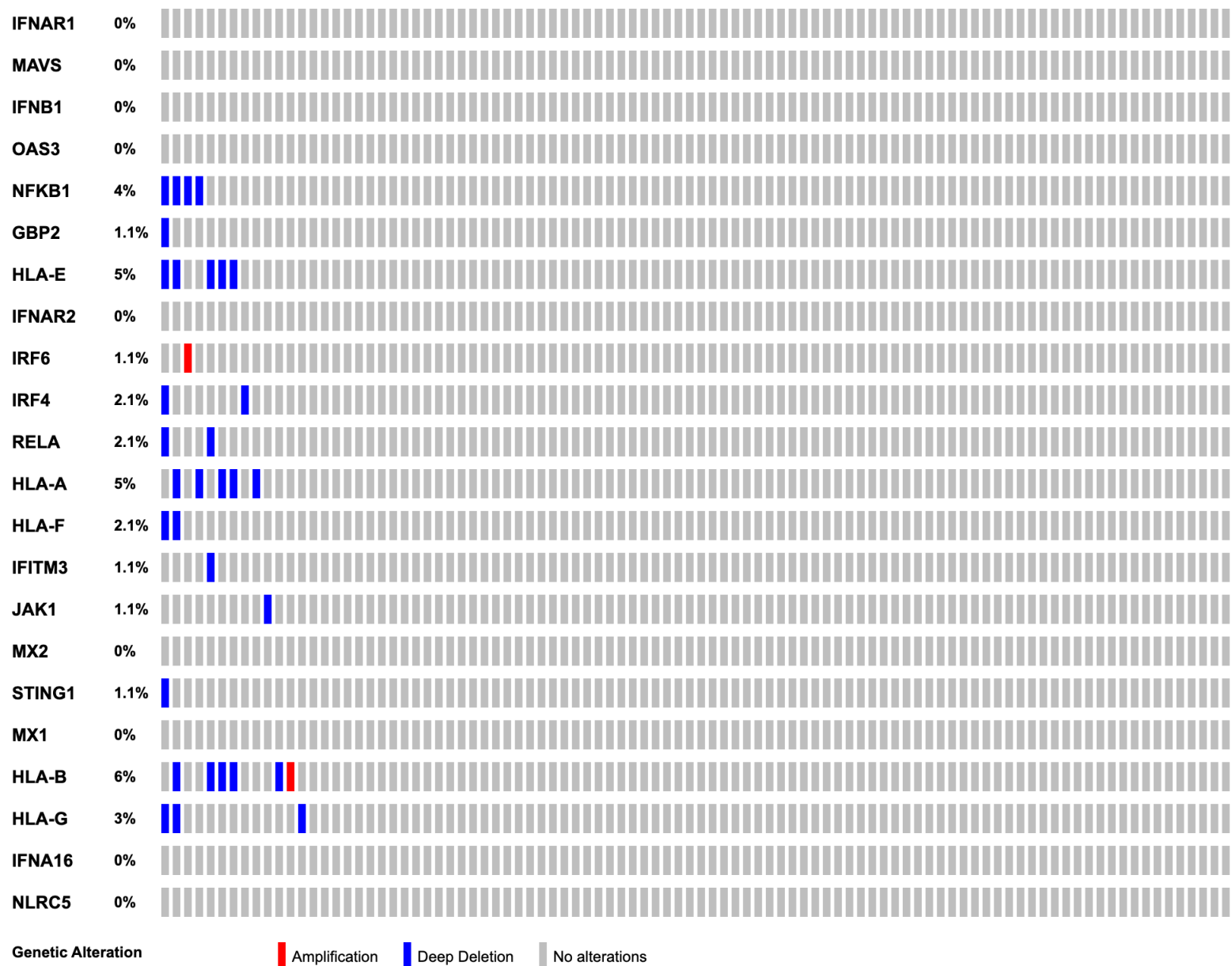

Supplement: Supporting Figure S12 [file mmc12.pdf]
