# Supplementary material for: Disruption of polyunsaturated fatty acid biosynthesis drives STING-dependent acute myeloid leukemia cell maturation and death
Source: J Biol Chem. 2024 Mar 22;300(5):107214. doi: 10.1016/j.jbc.2024.107214 (PMC11061745; doi:10.1016/j.jbc.2024.107214)
Supplement: Supporting Figure S8 [file mmc8.pdf]

Figure S8

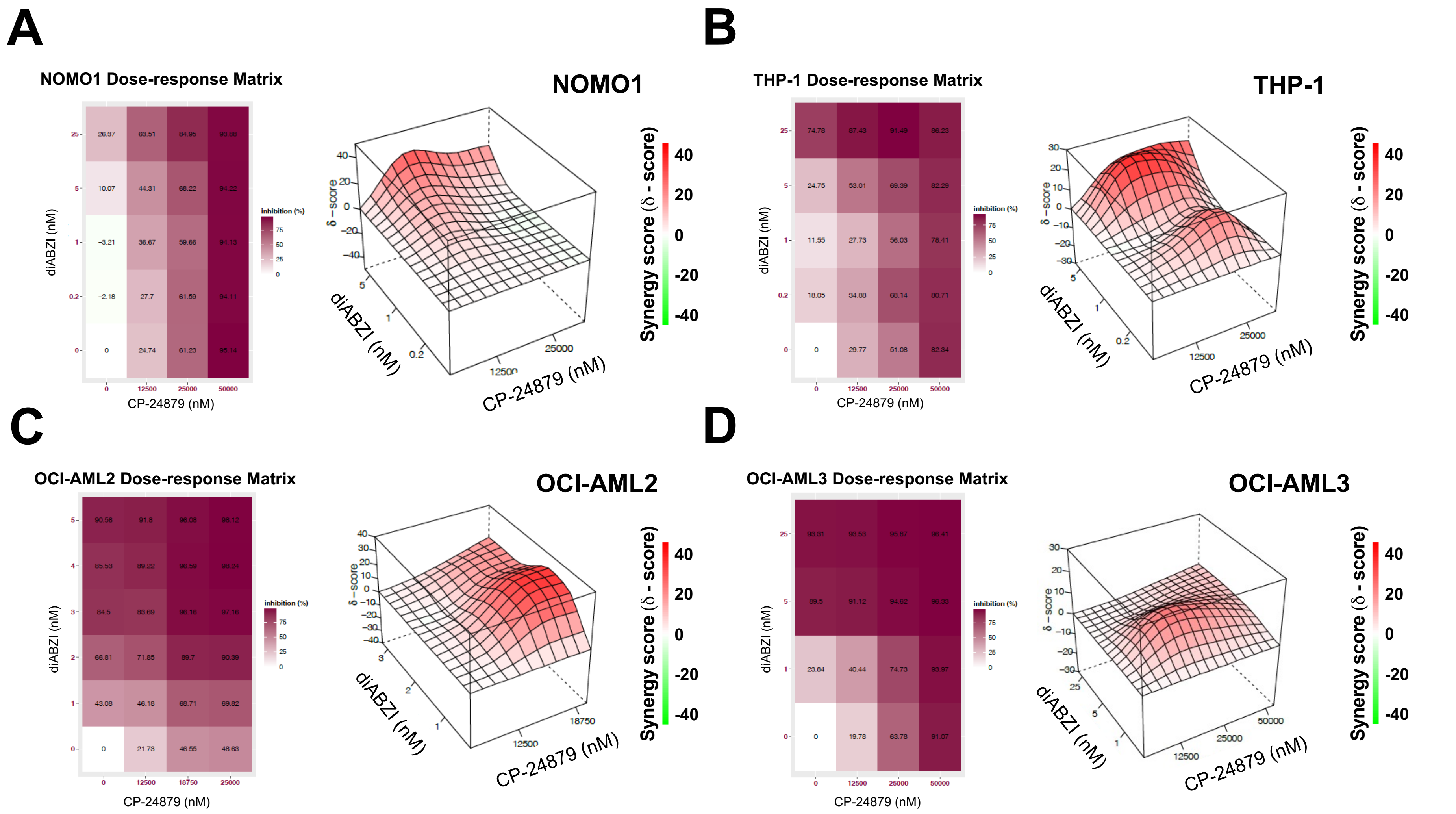
A
NOMO1 Dose-response Matrix
26.37 63.51 84.95 93.68
10.07 44.31 68.22 94.22
-3.21 36.67 59.66 94.13
-2.18 27.7 61.59 94.11
0 24.74 61.23 95.14
diABZI (nM)
CP-24879 (nM)
inhibition (%)
NOMO1
Synergy score (δ - score)
diABZI (nM)
CP-24879 (nM)
B
THP-1 Dose-response Matrix
74.78 87.43 91.49 86.23
24.75 53.01 69.39 82.29
11.55 27.73 56.03 78.41
18.05 34.88 68.14 80.71
0 29.77 51.08 82.34
diABZI (nM)
CP-24879 (nM)
inhibition (%)
THP-1
Synergy score (δ - score)
diABZI (nM)
CP-24879 (nM)
C
OCI-AML2 Dose-response Matrix
90.56 91.8 96.08 98.12
85.53 89.22 96.59 98.24
84.5 83.69 96.16 97.16
66.81 71.85 89.7 90.39
43.08 46.18 68.71 69.82
0 21.73 46.55 48.63
diABZI (nM)
CP-24879 (nM)
inhibition (%)
OCI-AML2
Synergy score (δ - score)
diABZI (nM)
CP-24879 (nM)
D
OCI-AML3 Dose-response Matrix
93.31 93.53 95.87 96.41
89.5 91.12 94.62 96.33
23.84 40.44 74.73 93.97
0 19.78 63.78 91.07
diABZI (nM)
CP-24879 (nM)
inhibition (%)
OCI-AML3
Synergy score (δ - score)
diABZI (nM)
CP-24879 (nM)
