# Supplementary material for: Disruption of polyunsaturated fatty acid biosynthesis drives STING-dependent acute myeloid leukemia cell maturation and death
Source: J Biol Chem. 2024 Mar 22;300(5):107214. doi: 10.1016/j.jbc.2024.107214 (PMC11061745; doi:10.1016/j.jbc.2024.107214)
Supplement: Supporting Figure S10 [file mmc10.pdf]

Figure S10

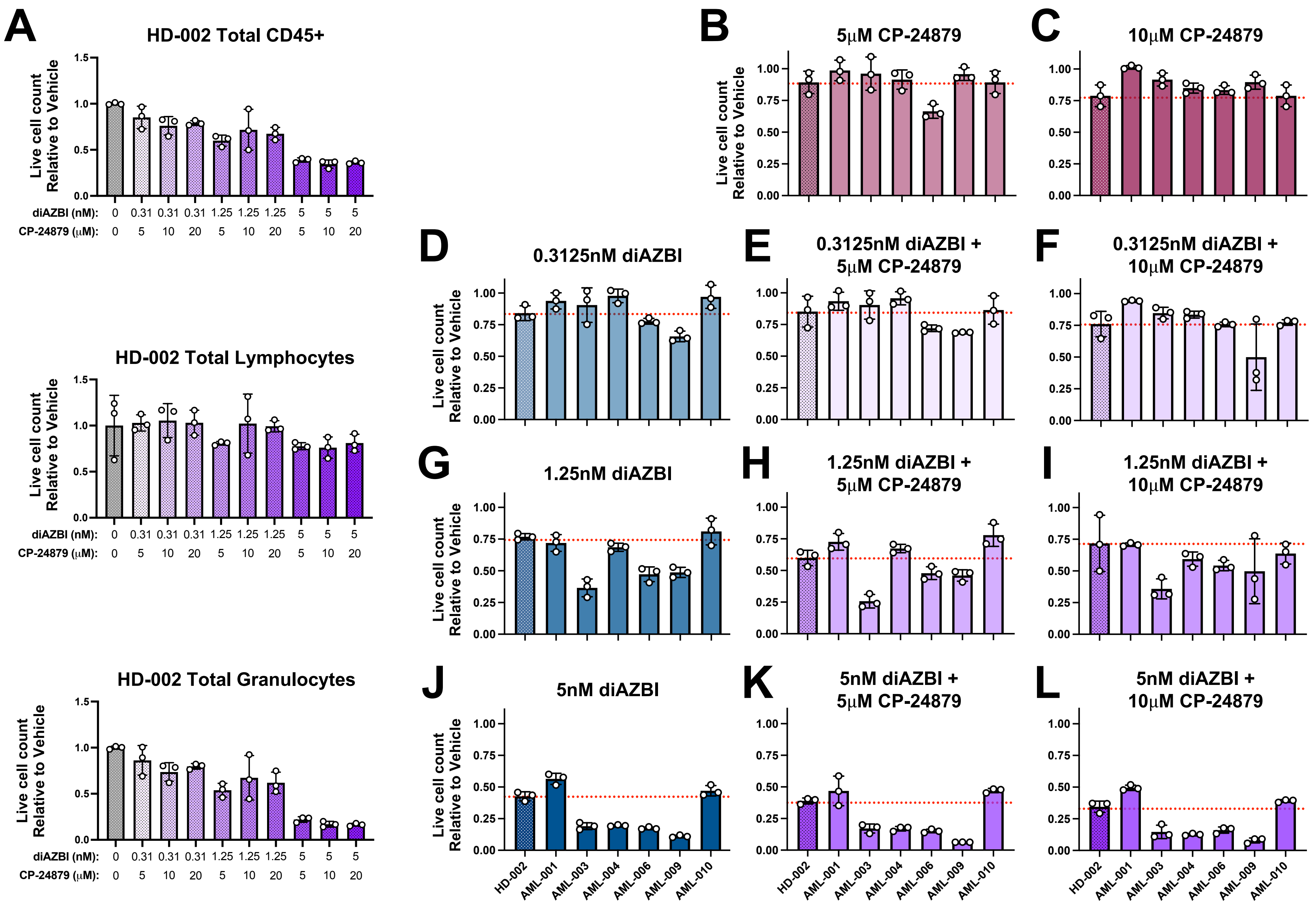
A
HD-002 Total CD45+
Live cell count Relative to Vehicle
HD-002 Total Lymphocytes
HD-002 Total Granulocytes
diAZBI (nM): 0 0.31 0.31 0.31 1.25 1.25 1.25 5 5 5
CP-24879 (μM): 0 5 10 20 5 10 20 5 10 20
B
5μM CP-24879
C
10μM CP-24879
D
0.3125nM diAZBI
E
0.3125nM diAZBI + 5μM CP-24879
F
0.3125nM diAZBI + 10μM CP-24879
G
1.25nM diAZBI
H
1.25nM diAZBI + 5μM CP-24879
I
1.25nM diAZBI + 10μM CP-24879
J
5nM diAZBI
K
5nM diAZBI + 5μM CP-24879
L
5nM diAZBI + 10μM CP-24879
HD-002 AML-001 AML-003 AML-004 AML-006 AML-009 AML-010
