# Supplementary material for: Disruption of polyunsaturated fatty acid biosynthesis drives STING-dependent acute myeloid leukemia cell maturation and death
Source: J Biol Chem. 2024 Mar 22;300(5):107214. doi: 10.1016/j.jbc.2024.107214 (PMC11061745; doi:10.1016/j.jbc.2024.107214)
Supplement: Supporting information [file mmc13.pdf]

Supplementary Information

DISRUPTION OF POLYUNSATURATED FATTY ACID BIOSYNTHESIS DRIVES STING-DEPENDENT ACUTE MYELOID LEUKEMIA CELL MATURATION AND DEATH

Joice Kanefsky[1#], Mary Basse[2#], Judith Sokei[2], Orsola di Martino[2], Liana Valin[2], Yorrick Jaspers[3], Esteban Martinez[1], Jacklyn Huhn[1], Daniela Di Marcantonio[1], Jeffrey A. Magee[2], Aaron R. Goldman[4], Hsin-Yao Tang[4], Francesca Ferraro[2], Stephan Kemp[3], David L. Wiest[1*+], Stephen M. Sykes[2*+]

[1] Fox Chase Cancer Center, Temple University Health System, Philadelphia, PA, USA

[2] Washington University in Saint Louis, School of Medicine, Department of Medicine, St. Louis, MO, USA

[3] Amsterdam University Medical Centers, Amsterdam, NH, Netherlands

[4] Proteomics & Metabolomics Facility, Wistar Institute, Philadelphia, PA, USA.

* s.m.sykes@wustl.edu

* david.wiest@fccc.edu

[#] These authors contributed equally to this work

[+] These authors contributed equally to this work

Gene.symbol	logFC	adj.P.Val
HACD1	2.90608033	8.30E-05
FADS1	2.73760524	1.96E-07
TECR	2.37430931	3.51E-07
ACOT7	1.68095802	3.05E-03
FASN	1.51918074	1.04E-04
ECHS1	1.3888164	6.17E-07
ACACA	1.17852022	4.97E-06
MCAT	1.14915748	1.89E-03
FADS2	1.08785083	1.26E-03
HSD17B12	1.0495052	8.34E-04
HACD2	1.03469773	1.40E-03
ECH1	0.98207123	2.04E-07
SCD	0.88420302	3.20E-01
ACAA2	0.74720751	5.03E-03
OXSM	0.63671344	9.41E-02
ELOVL6	0.5429308	3.74E-01
FADS3	0.54058976	4.89E-02
SCD5	0.525086	5.95E-01
ACLY	0.25916356	2.92E-01
ACSL3	0.25853365	3.29E-01
ACOT11	0.23521916	3.22E-01
ACOX3	0.21786374	2.86E-01
ELOVL1	0.12682143	6.88E-01
ACSL5	0.09982112	7.97E-01
ELOVL5	0.07340468	7.66E-01
ECHDC2	0.05665133	9.29E-01
ELOVL4	0.04786558	8.23E-01
ACSL6	0.02632591	7.55E-01
ACOX1	0.01249551	8.83E-01
ELOVL2	0.00788475	9.39E-01
BAAT	0.00360875	9.58E-01
OLAH	-0.0001645	9.98E-01
ECHDC3	-0.1563855	5.04E-01
ACAA1	-0.3578558	9.19E-02
ACSL1	-2.9955965	8.89E-06

Table S1. Fatty acid synthetic genes presented in figure 1B. Gene expression data collected from [1] via the Gene Expression Omnibus (Series GSE 9476, Platform GPL96).

Patient Sample	Tissue	Age	Sex	Dx	Genetics [VAF]
AML-001	BM	68	M	AML	CEBPA^{K352R} [42.6%]; DNMT3A$^{N501M;fs57*}$ [34.9%]; U2AF1$^{P301Q;fs44*}$ [81.6%]; UBA1^{Q157R} [42.6%]
AML-002	PB	69	*M*	MDS	PHF6^{G12D} [19.3%]; RAD21^{D61V} [2.9%]; SETBP1^{G199R} [40.3%]; SMC1A^{R625C} [46.1%]; SMC3^{R160H} [97.2%]; U2AF1^{R248Q} [96.2%]
AML-003	BM	60	F	AML	NOTCH1$^{I679D;fs21*}$ [2.1%]; SETBP1^{P113S} [47.4%]
AML-004	BM	86	M	AML	ASXL1$^{E635R;fs15*}$ [43.2%]; PHF6^{G12S} [6.0%] RAD21^{E76K} [14.2%]; SETBP1$^{S141A;142insDATF}$ [37.6%]
AML-005	BM	81	M	AML	FLT3$^{ITD\ (612insDDLKWEFPRENLEF)}$ [37.8%]; NPM1$^{W288C;fs12*}$ [42.2%];SMC1A^{R586Q} [46.1%]; TET2$^{S407Ffs20*/Q1138*}$ [45.8%;45.3%]
AML-006	BM	62	M	AML	DNMT3A^{R882H} [33.6%]; IDH2^{R127K} [22.1%]; SMC3^{K311E} [20.5%]
AML-007	BM	48	F	tAML	FLT3$^{D835Y/dupG583-D593}$ [20.6%/6.5%]; IDH2^{R140Q} [28.2%]; NRAS^{G13D} [2.1%];
AML-008	BM	52	F	AML	IDH2^{R140Q} [45.7%]; NRAS$^{W288C;fs12*}$ [43.0%];
AML-009	PB	71	F	AML	Complex Karyotype
AML-010	BM	60	F	AML	NOTCH1^{Y333*} [46.0%]; U2AF1^{R248Q} [77.3%]
HD-001	BM	42	F	N/A	N/A
HD-002	BM	35	F	N/A	N/A
HD-003	BM	34	F	N/A	N/A

Table S2. AML patient and healthy donor characteristics (MDS = Myelodysplastic Syndrome; tAML = therapy-related AML).

Gene \ Cell line	SKNO1	MOLM13	MOLM14	OCI-AML2	HEL	OCI-AML4	OCIM2	ME1	TF1	OCIM1	SET2	SIGM5	EOL1	CMK	PLB985	P31FUJ	KASUMI6	CMK115	GDM1
GBP2											✓								
HLA-A															✓				
HLA-B									✓										
HLA-E																		✓	
HLA-F													✓						
HLA-G				✓	✓														
IFITM3														✓					
IFNA16								✓											
IFNAR2																	✓		
IFNB1						✓													
IRF4																✓			
IRF6																		✓	
JAK1										✓									
MAVS							✓												
MX1											✓								
MX2					✓														
NFKB1																			✓
NLRC5	✓																		
OAS3		✓	✓																
RELA															✓				
STING1												✓							
IFNAR1				✓															

Table S3. Genes of the type-1 interferon pathway are shown in the first column and various AML cell lines are represented in the following columns. A shaded cell (pink) with a check mark signifies that given cell line contains a mutation in the select gene. All data was extracted from the DepMap Portal (https://depmap.org/portal/interactive/).

qPCR primers		
**Gene**		**Sequence 5' -> 3'**
IRF7	Forward	CCACGCTATACCATCTACCTGG
	Reverse	GCTGCTATCCAGGGAAGACACA
STAT2	Forward	CAGGTCACAGAGTTGCTACAGC
	Reverse	CGGTGAACTTGCTGCCAGTCTT
IFIT1	Forward	GCCTTGCTGAAGTGTGGAGGAA
	Reverse	ATCCAGGCGATAGGCAGAGATC
IFIT2	Forward	GGAGCAGATTCTGAGGCTTTGC
	Reverse	GGATGAGGCTTCCAGACTCCAA
IFIT3	Forward	CCTGGAATGCTTACGGCAAGCT
	Reverse	GAGCATCTGAGAGTCTGCCCAA
ISIG15	Forward	CTCTGAGCATCCTGGTGAGGAA
	Reverse	AAGGTCAGCCAGAACAGGTCGT
CXCL10	Forward	GGTGAGAAGAGATGTCTGAATCC
	Reverse	GTCCATCCTTGGAAGCACTGCA

Table S4. List of qPCR primers used throughout the study.

Supplemental Figure Legends

Figure S1. Genetic inhibition of FADS1 antagonizes the expansion of mouse and human AML cells *in vitro* and in *vivo*. (A) Comparison of FADS1 mRNA levels between AML cases bearing wild-type FLT3 (FLT3-WT) and FLT3 internal tandem duplication (FLT3-ITD). The data was extracted from two independent cohorts [2], [3] using the Leukemia Gene Atlas. **(B)** FACS-purified GFP+ MLL-AF9 cells expressing shNT or shFads1.2 were cultured in cytokine-enriched methylcellulose and colonies were enumerated after 7 days (****$p<0.0001$). **(C)** *In vitro* growth curve for mouse MLL-AF9 leukemia cells. Data points represent the fold change in GFP% over day 3 for each condition (MLL-AF9-shNT vs. shFads1.1: Day 5, ****$p<0.0001$; Day 7, ****$p<0.0001$; Day 10, ***$p=0.0003$; and MLL-AF9-shNT vs. shFads1.2: Day 7, ***$p=0.0003$; Day 10, ***$p=0.0004$). **(D)** FACS-purified GFP+ MLL-AF9 cells expressing shNT, shFads1.1 (*top panel*) or shFads1.2 (*bottom panel*) were cultured in cytokine-enriched methylcellulose with vehicle or 20μM Arachidonic Acid (AA). Colonies were then enumerated after 7 days (shFads1.1-vehicle vs. -20μM AA, p**=0.0065 and shFads1.2-vehicle vs. -20μM AA, p***=0.0004). **(E)** Flow cytometric analysis of MLL-AF9 bone marrow cells recovered from leukemic mice depicting the percentage of GFP+ (shRNA-expressing) cells per mouse (shNT vs. shFads1.1, $p=0.0111$ and shNT vs. shFads1.2, $p=0.019$). **(F & G)** Western blot analysis of FACS-purified (*F*) OCI-AML3 cells or (*G*) MOLM14 expressing control i-shNT, i-shFADS1.1, or i-shFADS1.2. **(H & I)** Relative growth of (*H*) OCI-AML3 and (*I*) MOLM14 single cell clones expressing i-shNT, human-targeting i-shFADS1.1 or i-shFADS1.2. Counting beads were used as reference during flow cytometric analysis at the indicated

time points (OCI-AML3: i-shNT vs. i-shFADS1.1, ****p<0.0001; i-shNT vs. i-shFADS1.2, **p=0.017; and MOLM14: i-shNT vs. i-shFADS1.1, **p=0.0039; i-shNT vs. i-shFADS1.2, **p=0.0034). Dots represent individual data points and error bars represent SD.

Figure S2. FADS1 knockdown leads to AML cell cycle arrest, maturation and death. **(A-B)** MOLM14 (A) or OCI-AML3 (B) expressing i-shNT or i-shFADS1.1 were analyzed for %Annexin V^+ by flow cytometry, 5 days post-DOX (MOLM14: i-shNT vs. i-shFADS1.1, **p=0.0034; i-shNT vs. i-shFADS1.2, ****p<0.0001; and OCI-AML3: i-shNT vs. i-shFADS1.1, **p=0.0094). **(C-D)** Flow cytometric analysis of MOLM14 (C) and OCI-AML3 (D) single cell clones for the expression of the myeloid marker CD11b by flow cytometry, 5 days post-DOX (MOLM14: i-shNT vs. i-shFADS1.1, ****p<0.0001; i-shNT vs. i-shFADS1.2, **p=0.0094; and OCI-AML3: i-shNT vs. i-shFADS1.1, ****p<0.0001). **(E)** Wright-Giemsa staining of MOLM14 cells expressing i-shNT, i-shFADS1.1, or i-shFADS1.2 shRNAs at 5 days post doxycycline induction (20x magnification). **(F)** Percent of internalized fluorescently labeled (PE) *E. coli* peptides by NOMO1 cells expressing i-shNT or i-shFADS1.2. Measurements were taken on day 5 post-doxycycline induction (i-shNT vs. i-shFADS1.2 at 37°C, ***p=0.0003). **(G)** Wright-Giemsa staining of FACS-purified MLL-AF9 cells expressing shNT or shFads1.1 (100x magnification). **(H)** Percent of internalized fluorescently labeled (PE) *E. coli* peptides by MLL-AF9 cells expressing shNT or shFads1.2 as measured by flow cytometry at day 9 days post-transduction (shNT vs. i-shFads1.2 at 37°C, **p=0.0014).

Figure S3. FADS1 inhibition selectively depletes 20:4 fatty acid in storage lipids. **(A)** Ratios of total signals from phospholipids containing 20:4 or 20:3 FA in the sn-2 position for MOLM14 cells (p=0.0005). **(B)** Analysis of fatty acid composition for shFADS1 vs. control shNT cells based on the total MS signal (lipid abundance) with the specified total degree of unsaturation for MOLM14 cells. **(C)** Total signal intensity (*left panel*) and signal intensity for Cholesteryl Esters containing a 20:4 fatty acid in the sn-2 position (*right panel*) for NOMO1 and MOLM14 cells (NOMO1, i-shNT vs. i-shFADS1.1, total ChE, **p=0.007; i-shNT vs. i-shFADS1.2, total ChE, ***p=0.0003; i-shNT vs. i-shFADS1.1, 20:4 *sn-2* ChE, *p=0.0165; and MOLM14, i-shNT vs. i-shFADS1.1, 20:4 *sn-2* ChE, *p=0.0342). **(D & E)** Total signal intensity (*left panel*), signal intensity for Triglycerides containing a 20:4 fatty acid (*middle panel*) or a 20:5 fatty acid (*right panel*) in the sn-2 position for (*D*) NOMO1 (i-shNT vs. i-shFADS1.1: total TG, ****p<0.0001; 20:4 TG, ****p<0.0001; 20:5 TG, ****p<0.0001; and i-shNT vs. i-shFADS1.2: 20:4 TG, **p=0.0028; 20:5 TG, **p=0.0038) and (*E*) MOLM14 cells (i-shNT vs. i-shFADS1.1: ***total TG, p=0.0002; 20:4 TG, *p=0.0391; 20:5 TG, *p=0.0257).

Figure S4. FADS1 inhibition drives STING activation. (A) Heatmap representation of the expression of genes identified to be differentially regulated between i-shNT and i-shFADS1.1 in the 'Response to Virus (GO_UP)' signature in the top panel of Fig 5B. **(B)** qPCR analysis of the specified genes in i-shNT- and i-shFADS1.1-expressing MOLM14 cells at 40h post-doxycycline induction (i-shNT vs i-shFADS1.1: *IFIT1*, ****p<0.0001; *IFIT2*, ****p<0.0001; *IFIT3*, ****p<0.0001; *CXCL10*, **p=0.0024; *IRF7*, ***p=0.0001;

STAT2, ***p=0.0003). **(C)** Cytoplasmic extracts from MOLM14-expressing i-shNT or i-shFADS1.1 and treated with DOX 40 hours earlier were subjected to enzyme-linked immunoabsorbent assay (ELISA) to detect cGAMP levels (p=0.0069). **(D)** Gene Set Enrichment Analysis (GSEA) enrichment score curve of Toll-Like Receptor cascade genes in NOMO1 cells expressing i-shFADS1.1 vs i-shNT control. The analysis was performed using the Reactome Pathway Database. **(E)** Western blot analysis of NOMO1 (*left panel*) and MOLM14 (*right panel*) cells expressing i-shNT or i-shFADS1.1 with the indicated antibodies. The cells were harvested at 40h post-doxycycline induction. **(F)** Western blot analysis of THP-1 (*right panel*) and NOMO1 (*left panel*) cells co-expressing Cas9 and control (STING^{WT}) or STING-targeting CRISPR guides (STING^{KO}) with the indicated antibodies. **(G)** THP-1 STING^{WT} (i.e. wild-type) and STING^{KO} cells expressing i-shNT, i-shFADS1.1 or i-shFADS1.2 were analyzed for CD11b MFI (i-shNT vs i-shFADS1.1, ****p<0.0001; i-shNT vs i-shFADS1.2, **p=0.003) using flow cytometry 4 days post-DOX induction. **(H)** cDNA recovered from THP-1-STING^{WT}- and -STING^{KO} cells expressing i-shNT or shFads1.1 were analyzed by qPCR for the expression of the indicated genes. The data is presented as the fold change (FC) in gene expression of i-shFADS1.1-expressing cells over i-shNT-expressing cells in each cellular genotype (STING^{WT} vs STING^{KO}: *IFIT1*, ****p<0.0001; *IFIT2*, ****p<0.0001; *IFIT3*, ****p<0.0001; *CXCL10*, *p=0.012; *IRF7*, ****p<0.0001; *ISIG15*, ***p=0.0007).

Figure S5. Pharmacological inhibition of FADS enzymes imparts anti-AML activity.

(A) MLL-AF9 cells (*left panel*) and HSPCs (*right panel*) were treated with the indicated

concentrations of CP-24879 for 24 hours, washed and then plated in cytokine-enriched methylcellulose (10,000 for HSPCs or 500 MLL-AF9). Colonies were enumerated 7 days after plating (MLL-AF9: 0 vs. 25μM, *p=0.0206; and 0 vs. 50μM, ***p=0.0009). **(B)** NOMO1 were treated with the indicated concentrations of CP-24879 for 48 hours and then assessed by flow cytometry for CD11b MFI (*left panel,* all comparisons, ****p<0.0001) or % Annexin V^+ (*middle panel*: 0 vs. 12.5μM, **p=0.0066; 0 vs. 25μM, **p=0.0012; and 0 vs. 50μM, ****p<0.0001). *Right panel*, vehicle and 25μM CP-24879-treated cells were subjected to Wright-Giemsa staining at 48h (100x magnification). **(C)** THP1 were treated with the indicated concentrations of CP-24879 for 48 hours and then assessed by flow cytometry for CD11b MFI (*left panel:* 0 vs. 12.5μM, ***p=0.0007; 0 vs. 25μM, ****p<0.0001; and 0 vs. 50μM, ****p<0.0001) or % Annexin V^+ (*middle panel*: 0 vs. 25μM, *p=0.0294; and 0 vs. 50μM, ****p<0.0001). *Right panel*, vehicle and 25μM CP-24879-treated cells were subjected to Wright-Giemsa staining at 48h (100x magnification). **(D)** THP-1 $STING^{WT}$ and $STING^{KO}$ cells were treated with the indicated concentration of CP-24978 for 48 hours and then analyzed for %Annexin V^+ by flow cytometry ($STING^{WT}$ vs. $STING^{KO}$: 20μM vs. 20μM, ***p=0.0002; 40μM vs. 40μM, ***p=0.0006). **(E)** cDNA recovered from THP-1-$STING^{WT}$- and -$STING^{KO}$ cells treated with vehicle or 40μM CP-24879 were analyzed by qPCR for the expression of the indicated genes. The data is presented as the fold change (FC) in gene expression of CP-24879-treated cells over vehicle-treated cells in each cellular genotype ($STING^{WT}$ vs $STING^{KO}$: *IFIT1*, *p=0.0158; *IFIT2*, **p=0.003; *IFIT3*, **p=0.0011; *CXCL10*, *p=0.016; *IRF7*, ****p<0.0001; *ISIG15*, **p=0.0086). **(F-H)** The indicated cells lines were treated with either increasing concentrations of CP-24879

or sc-26126 for 48 hours and then assessed for % Annexin V+ by flow cytometry: (*F*) NOMO1 (CP-24879 vs. sc-26126: 40μM vs. 40μM, ***p=0.0006); (*G*) MOLM14 (CP-24879 vs. sc-26126: 20μM vs. 20μM, **p=0.0103); (*H*) THP-1 (CP-24879 vs. sc-26126: 10μM vs. 10μM, *p=0.0111; 20μM vs. 20μM, ***p=0.0004; 40μM vs. 40μM, ***p=0.0009). **(I-K)** The indicated cells lines were treated with either increasing concentrations of CP-24879 or sc-26126 for 48 hours and then assessed for CD11b MFI by flow cytometry: (*I*) NOMO1 (CP-24879 vs. sc-26126: 10μM vs. 10μM, *p=0.0281; 20μM vs. 20μM, ***p=0.0008; 40μM vs. 40μM, ***p=0.0004); (*J*) MOLM14 (CP-24879 vs. sc-26126: 20μM vs. 20μM, *p=0.0105; 40μM vs. 40μM, ****p<0.0001); (*K*) THP-1 (CP-24879 vs. sc-26126; 20μM vs. 20μM, **p=0.0086; 40μM vs. 40μM, ***p=0.0001).

Figure S6. Pharmacological inhibition of FADS enzymes reduced live cells of patient-derived AML samples but not that healthy BM-derived hematopoietic cells. (A-F) BM or PB samples recovered from patients diagnosed with AML were treated with the indicated concentrations of CP-24879 for 4 days and then analyzed by flow cytometry to count live AML cells. (*A*) AML-005: Live cell counts, 0 vs. 10μM, *p=0.0191 and 0 vs. 20μM, **p=0.0017. (*B*) AML-006: Live cell counts, 0 vs. 5μM, *p=0.0139 and 0 vs. 20μM, **p=0.005. (*C*) AML-007: Live cell counts, 0 vs. 10μM, **p=0.0028 and 0 vs. 20μM, ***p=0.0004. (*D*) AML-008: Live cell counts, 0 vs. 10μM, *p=0.0264 and 0 vs. 20μM, **p=0.0055. (*E*) AML-009: No significant differences. (*F*) AML-010: Live cell counts, 0 vs. 10μM, *p=0.0216 and 0 vs. 20μM, ***p=0.0005. **(G)** BM samples recovered from healthy donor #3 were treated with the indicated concentrations of CP-24879 for 4 days and then

analyzed by flow cytometry to count live cells. Dots represent individual data points and error bars represent SD.

Figure S7. STING agonism displays anti-leukemia activity but also reduces live granulocyte counts. (A) THP-1-STING^{WT} and -STING^{KO} were treated with the indicated concentrations of diAZBI for 48 hours and then assessed for live cells (PI^{-}) by flow cytometry. **(B-G)** BM or PB samples recovered from patients diagnosed with AML were treated with the indicated concentrations of diAZBI for 4 days and then analyzed by flow cytometry to count live AML cells. (*B*) AML-005: Live cell counts, 0 vs. 5μM, ***p=0.0008, 0 vs. 10μM, ***p=0.0008 and 0 vs. 20μM, ***p=0.0007. (*C*) AML-006: Live cell counts, 0 vs. 10μM, **p=0.0014 and 0 vs. 20μM, ****p<0.0001. (*D*) AML-007: No significant differences. (*E*) AML-008: Live cell counts, 0 vs. 5μM, ***p=0.0009, 0 vs. 10μM, ***p=0.0006 and 0 vs. 20μM, ***p=0.0007. (*F*) AML-009: Live cell counts, 0 vs. 5μM, **p=0.004, 0 vs. 10μM, ***p=0.0009 and 0 vs. 20μM, ****p<0.0001. (*G*) AML-010: Live cell counts, 0 vs. 10μM, *p=0.0494 and 0 vs. 20μM, ***p=0.0002. **(H)** BM samples recovered from healthy donors were treated with the indicated concentrations of diAZBI for 4 days and then analyzed by flow cytometry to count live CD45+ cells. *Left panel*, HD-001. *Middle panel*, HD-002: 0nM vs. 0.312nM, *p=0.0101; 0nM vs. 1.25nM, ***p=0.0001; 0nM vs. 5nM, ****p<0.0001). *Right panel*, HD-003: 0nM vs. 0.1nM, *p=0.0466; 0nM vs. 1nM, *p=0.0126; 0nM vs. 10nM, **p=0.0085). **(I)** BM samples recovered from healthy donors were treated with the indicated concentrations of diAZBI for 4 days and then analyzed by flow cytometry to count CD14^{+}CD16^{+} granulocytes. *Left panel*, HD-001: 0nM

vs. 10nM, *p=0.0149. *Middle panel*, HD-002: 0nM vs. 0.312nM, *p=0.0101; 0nM vs. 1.25nM, ***p=0.0001; 0nM vs. 5nM, ****p<0.0001). *Right panel*, HD-003: 0nM vs. 1nM, *p=0.0357; 0nM vs. 10nM, *p=0.0285). **(J)** BM samples recovered from healthy donors were treated with the indicated concentrations of diAZBI for 4 days and then analyzed by flow cytometry to count CD19+ Lymphocytes. *Left panel*, HD-001: 0nM vs. 0.1nM, *p=0.0281. *Middle panel*, HD-002. *Right panel*, HD-003.

Figure S8. Pharmacological blockade of FADS cooperates with STING agonism on human AML cell lines. (A-D) (*A*) NOMO1, (*B*) THP-1, (*C*) OCIAML2 and (*D*) OCI-AML3 cells were treated with the indicated concentrations of diAZBI and CP-24879. *Left panels* represent two-dimensional dose-response matrices indicating the percentage of inhibition of each combination of diAZBI (*y*-axis) and CP-24879 (*x*-axis). *Right panels* represent three-dimensional plots of the synergy/cooperation scores (*y*-coordinate) for each cell line treated with the indicated concentrations of STING agonist, diAZBI (*x*-coordinate) and CP-24879 (*z*-coordinate).

Figure S9. STING agonism variably cooperates with FADS1 inhibition on patient-derived AML samples. (A) Three dimensional plots of the synergy/cooperation scores (*y*-coordinate) for the indicated patient-derived AML samples treated with varying combinations of the STING agonist, diAZBI (*z*-coordinate) and CP-24879 (*x*-coordinate).

(B) Two-dimensional dose-response matrices indicating the percentage of inhibition of each combination of diAZBI (*x*-axis) and CP-24879 (*y*-axis) for each of the indicated patient-derived AML samples.

Figure S10. STING agonism may have a potential therapeutic window in AML. (A) BM cells from HD-002 were treated with the indicated concentrations of diAZBI and CP-24879. *Top panel,* data are presented as the ratio of total live cell counts in each condition divided by the average cell count of vehicle-treated cells. *Middle panel,* data are presented as a ratio of total lymphocyte counts in each condition divided by the average cell count of vehicle-treated cells. *Bottom panel,* data are presented as a ratio of total granulocyte counts in each condition divided by the average cell count of vehicle-treated cells. **(B-L)** BM cells from HD-002 or the indicated AML patients were treated with the indicated concentrations of diAZBI or CP-24879 and the data in each panel is presented as the ratio of drug-treated to vehicle-treated live cell counts for each sample. The indicated samples were treated with the indicated concentrations of: (*B-C*) CP-24879; (*D, G* and *J*) diAZBI; or (*E, F, H, I, K* and *L*) CP-24879 and diAZBI.

Figure S11. FADS1 inhibition disrupts FA monosaturation and reduces the expression of other enzymatic regulators of of FA synthesis. (A) Heatmap of total MS signals from 16:1 (*top panel*) and 18:1 (*bottom panel*) FA-containing lipid species. CL – Cardiolipin, SM – Sphingomyelin, ChE – Cholesteryl Ester, PA – Phosphatidic acid, PI – Phosphatidylinositol, PS – Phosphatidylserine, TG – Triglyceride, PE –

Phosphatidylethanolamine, PG – Phosphatidylglycerol, PC – Phosphatidylcholine, DG – Diglyceride, AcCa – Acyl carnitine, Cer – Ceramide, Hex1Cer – Hexosyl Ceramide with 1 hexose, Hex2Cer – Hexosyl Ceramide with 2 hexoses. **(B)** qPCR analysis of NOMO1 single cell clones for the specified genes: ****p<0.0001 for all indicated comparisons. **(C)** Same as in (B) but for MOLM14 cells: i-shNT vs. i-shFADS1, *FASN*, ***p=0.0002, *FADS1*, ***p=0.0002; i-shNT vs. i-shFADS1, *FASN*, **p=0.0055; ****p<0.0001 for all other indicated comparisons.

Figure S12. Summary of genes related to Type-I interferon Signaling that are mutated in pediatric AML. (A) Landscape of genetic aberrations in type-1 IFN genes from pediatric AML patients. Each bar represents a patient sample. All data was extracted from the TARGET AML Initiative via cBioPortal (https://www.cbioportal.org/).

Supplemental References

[1] D. L. Stirewalt *et al.*, "Identification of genes with abnormal expression changes in acute myeloid leukemia," *Genes Chromosomes Cancer*, vol. 47, no. 1, pp. 8–20, Jan. 2008, doi: 10.1002/gcc.20500.

[2] P. J. M. Valk, R. Delwel, and B. Löwenberg, "Gene expression profiling in acute myeloid leukemia," 2005.

[3] "Genomic and Epigenomic Landscapes of Adult De Novo Acute Myeloid Leukemia," *New England Journal of Medicine*, vol. 368, no. 22, pp. 2059–2074, May 2013, doi: 10.1056/NEJMoa1301689.
